# A comprehensive bibliometric analysis of ferroptosis in tumor resistance: development and emerging trends

**DOI:** 10.3389/fimmu.2025.1580222

**Published:** 2025-05-09

**Authors:** Liangyun Xie, Yafei Zhang, Xiedong Niu, Yuan Kang, Wenyao Li, Jun Yao

**Affiliations:** The First Affiliated Hospital, and College of Clinical Medicine of Henan University of Science and Technology, Luoyang, China

**Keywords:** ferroptosis, bibliometric analysis, tumor resistance, oxidative stress, tumor microenvironment

## Abstract

**Background:**

Ferroptosis is a regulated form of cell death characterized by iron dependency, lipid peroxidation, and oxidative stress. Since its discovery in 2012, ferroptosis has attracted significant interest for its potential to counteract tumor resistance across various therapeutic modalities, including chemotherapy, radiotherapy, immunotherapy, and targeted therapy. Despite notable progress, a systematic understanding of its underlying molecular mechanisms and translational potential remains underdeveloped, thus necessitating a comprehensive bibliometric analysis.

**Methods:**

We employed bibliometric tools, including VOSviewer, CiteSpace, and bibliometric.com, to analyze 2,663 articles related to ferroptosis and tumor resistance indexed in the Web of Science Core Collection from 2014 to 2024. The analysis included co-occurrence, co-citation, and clustering techniques to explore trends, influential keywords, prominent journals, leading institutions, and key contributors. Citation burst detection and temporal analysis were used to uncover emerging research hotspots and track the field’s evolution.

**Results:**

Over the past decade, the volume of publications in this field has grown rapidly, with China and the United States leading in both research output and academic influence. Notable institutions such as Central South University and Fudan University contributed significantly, while Kang Rui and Tang Daolin emerged as prolific authors. Key research hotspots identified include oxidative stress, tumor microenvironment, and nanomedicine, with emerging themes such as immunotherapy and autophagy gaining prominence. Temporal trends indicated a shift from mechanistic studies toward translational applications, emphasizing the integration of ferroptosis in clinical strategies to address tumor resistance.

**Conclusions:**

This bibliometric analysis highlights ferroptosis as a rapidly evolving field with significant contributions to understanding tumor resistance mechanisms. The identification of emerging themes and promising research directions offers valuable insights for future investigations and clinical applications of ferroptosis in overcoming tumor resistance.

## 1 Introduction

Ferroptosis, an iron-dependent form of regulated cell death characterized by lipid peroxidation and oxidative stress, is a novel mechanism that is significantly different from traditional forms of death such as apoptosis and necrosis. This concept was originally discovered by Brent R. Stockwell’s team at Columbia University in a study of RAS-mutated tumor cells and proposed in 2012 (1, 2). This novel iron-dependent cell death was named ferroptosis because the removal of iron ions effectively impaired the killing effect of erastin on tumor cells, whereas the removal of other metal ions had no such effect. The molecular mechanism of ferroptosis is mainly regulated by glutathione peroxidase 4 (GPX4), cysteine transporter (SLC7A11), and polyunsaturated fatty acid metabolism-related molecules, such as ACSL4 (3). These molecules are important for redox homeostasis and lipid metabolism in tumor cells, thereby highlighting its role in tumorigenesis and therapy resistance.

A major challenge in tumor therapy is the development of drug resistance, including chemoresistance, targeted therapy resistance, multidrug resistance (MDR), radiotherapy resistance, and immunotherapy resistance (4–6). Chemoresistance and targeted therapy resistance are mostly driven by mechanisms such as oxidative stress adaptation, lipid metabolism disorders, and overexpression of MDR genes (7–10), whereas radiotherapy resistance is often achieved through enhanced DNA repair, upregulation of antioxidant defenses, or tumor microenvironment (TME) hypoxia (11–13). Immunotherapeutic resistance, on the other hand, involves overexpression of immune checkpoint molecules (e.g., PD-L1), immunosuppressive effects of the tumor microenvironment (e.g., enrichment of M2-type macrophages and Treg cells), and reduced expression of tumor antigens (14, 15). Together, these resistance mechanisms limit the effectiveness of tumor treatment strategies. In recent years, the study of ferroptosis has provided a novel perspective on overcoming tumor drug resistance. Studies have shown that by inducing ferroptosis, the oxidative balance of tumor cells can be disrupted, the lipid metabolism status can be altered, and the expression of MDR genes can be inhibited, thus significantly improving the efficacy of chemotherapy and targeted therapy (16–18). In addition, ferroptosis improves tumor sensitivity to radiotherapy by enhancing the oxidative stress damage induced by radiotherapy (19). At the same time, its induced immunogenic cell death (ICD) promotes the release of antigens and activation of the tumor immune microenvironment, thus enhancing the efficacy of immunotherapy (20, 21). Ferroptosis inducers have shown significant potential in modulating these mechanisms, further highlighting the importance of ferroptosis in basic research and clinical translation (22, 23). Although the study of ferroptosis shows great potential in tumor therapy and overcoming drug resistance, there are still many questions about its molecular mechanisms, regulatory networks, and clinical translational applications that need to be addressed. In particular, research is still in its infancy on the interaction between TME, immune regulation, and iron death, as well as the clinical effects of iron death combination therapy. With the rapid increase in the number of studies in recent years, it is necessary to conduct a comprehensive and systematic review of the current status and development trend of iron death in tumor therapy and overcoming drug resistance.

Bibliometric analysis is a method based on quantitative data, which can reveal the hotspots, trends, and patterns of cooperation in the research field by analyzing indicators such as the number of publications, co-occurrence of keywords, and co-cited literature in academic literature, and provide systematic support for scientific research (24). In this study, based on the Web of Science core ensemble database, we searched the literature related to iron death and tumor drug resistance from 2014 to 2024, and used VOSviewer, CiteSpace, and bibliometric.com to perform co-occurrence analysis and cluster analysis to systematically explore the hotspots in the field, high-impact authors and institutions, research topics evolutionary trajectories, and future directions. To the best of our knowledge, this study represents the first comprehensive bibliometric analysis focusing specifically on ferroptosis and tumor resistance, aiming to bridge the knowledge gap between basic mechanisms and translational applications.

## 2 Materials and methods

### 2.1 Data retrieval and collection

**Figure 1:**
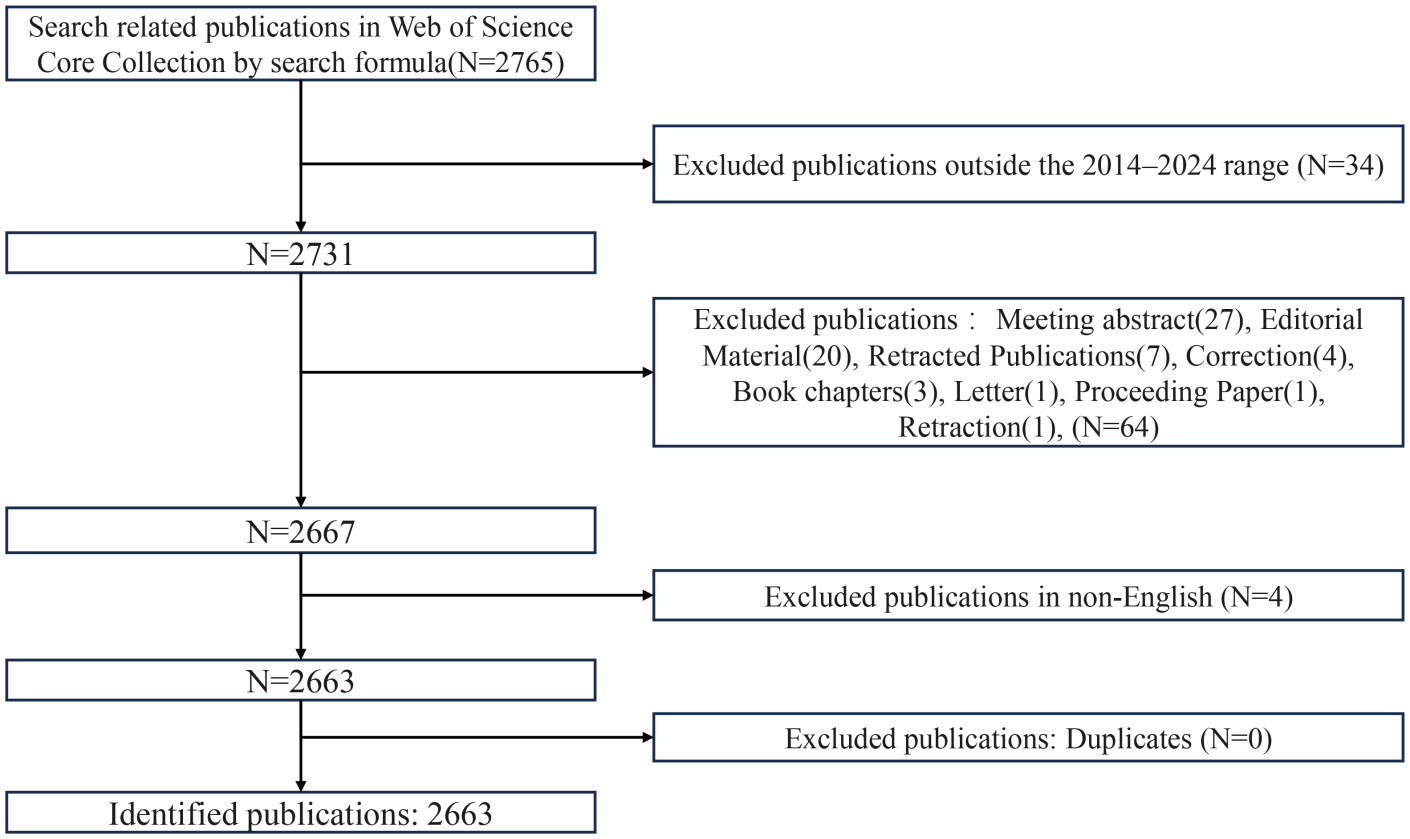
Flowchart of publication screening and selection process.

The bibliometric data for this study were sourced from the Web of Science Core Collection (WoSCC) database, which includes the Science Citation Index Expanded (SCIE), Social Sciences Citation Index (SSCI), and Emerging Sources Citation Index (ESCI). The data retrieval was conducted on January 2, 2025, and covered the period from January 1, 2014, to December 31, 2024, to ensure comprehensive coverage of research related to ferroptosis in tumor resistance. The search query was formulated as follows: TS=((“Ferroptosis” OR “Iron-dependent cell death” OR “GPX4” OR “SLC7A11” OR “ACSL4” OR “FSP1” OR (“Lipid peroxid*” AND “GPX4”) OR (“Oxidative stress” AND “SLC7A11”)) AND (“Cancer” OR “Tumor” OR “Carcinoma”) AND (“Chemoresist*” OR “Drug resist*” OR “Multidrug resist*” OR “Chemotherapy resist*” OR “Chemo-refractor*” OR “Sensiti*” OR “Chemo-sensiti*” OR “Drug sensiti*” OR “resensiti*” OR “Overcoming resist*”)). An initial search yielded 2,765 publications. After applying inclusion criteria based on document type (restricted to “articles” and “reviews”) and language (limited to “English”), a total of 2,663 publications were selected for bibliometric analysis. The data were exported in “plain text” format with “full records and cited references” for subsequent analysis. The final dataset was processed and analyzed using CiteSpace and VOSviewer. A detailed workflow of the selection and filtering process is provided in **Figure 1**.

### 2.2 Data analysis

In this study, bibliometric analysis was conducted using multiple tools to explore the research landscape of ferroptosis in tumor resistance. VOSviewer (version 1.6.20; Van Eck & Waltman, 2010) was used to construct co-occurrence networks and generate keyword co-occurrence density maps (25). The full counting method and default resolution (1.0) were applied. CiteSpace (version 6.4.R1, 64-bit; Chen, 2006) was used for burst detection analysis, dual-map overlay visualization, keyword clustering, and timeline analysis (26). The parameters were set as follows: time slicing from 2014 to 2024 (1 year per slice), node type depending on analysis objective, selection criteria based on g-index (k = 25), Cosine similarity for link strength, and Pathfinder pruning was applied. The clustering quality was assessed by Modularity Q = 0.3087 and Mean Silhouette S = 0.5347, indicating moderate structural significance and acceptable cluster consistency. Bibliometric.com was used to perform statistical analyses of publication outputs by country/region. These tools facilitated an in-depth exploration of collaborative patterns and emerging trends, offering a systematic understanding of ferroptosis and tumor resistance research.

## 3 Results

### 3.1 Annual publication trends

**Figure 2:**
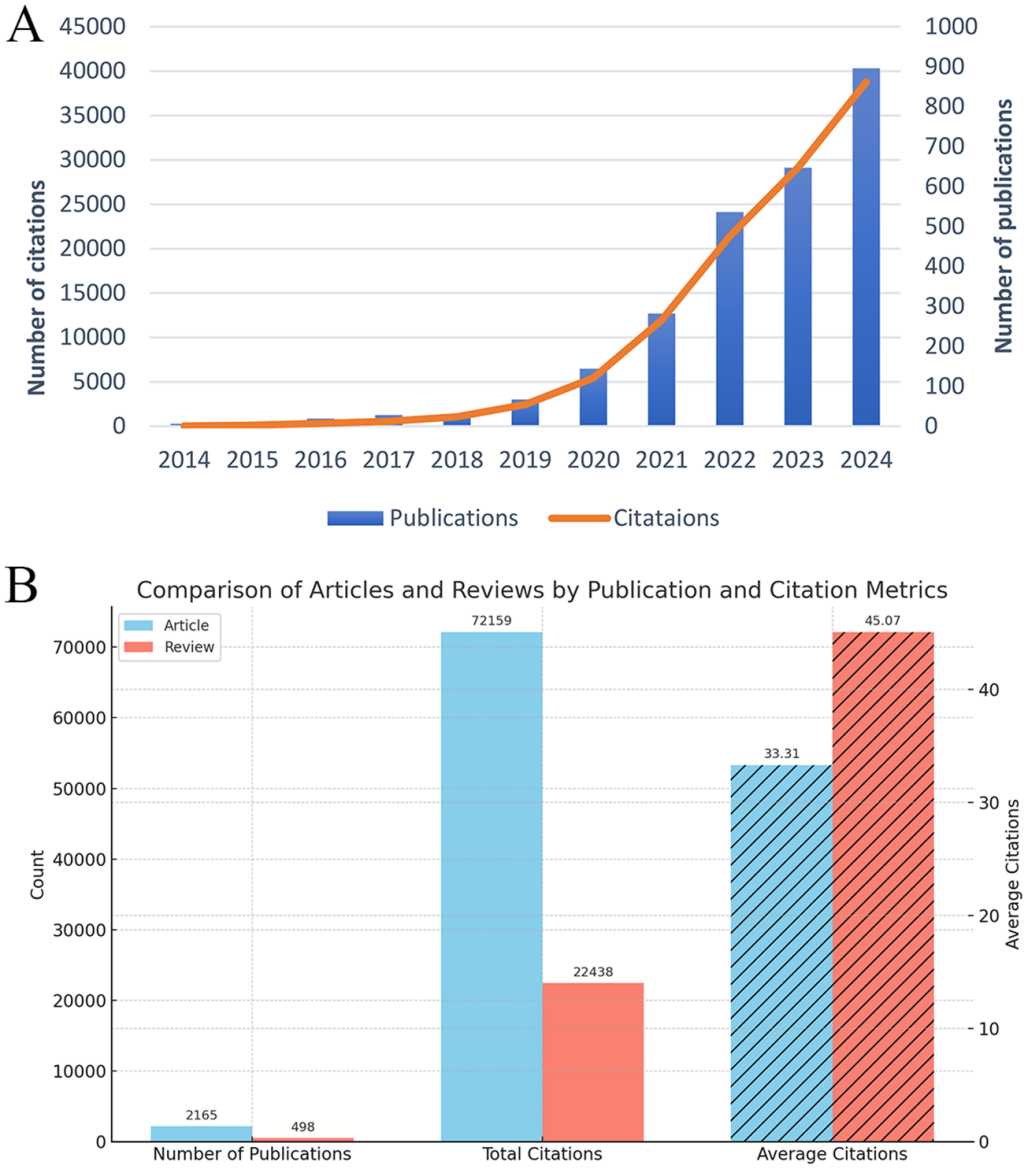
Trends and characteristics of publications on ferroptosis and tumor resistance: **(A)** Annual trends in publications and citations (2014–2024); **(B)** Comparison of articles and reviews by publication and citation metrics.

A total of 2,663 publications were included in this analysis, of which 81.3% were original articles and 18.7% were reviews (**Figure 2**). The annual publication volume in this field has shown remarkable growth over the past decade. In 2014, only six publications were identified, with a total of 27 citations. By 2024, the number of annual publications had surged to 897, accompanied by a citation count reaching 38,767. This exponential increase, particularly since 2019, underscores the growing recognition of ferroptosis as a pivotal research focus in the field of tumor resistance. As illustrated in **Figure 2A**, both publication output and citation volume have exhibited an exponential growth pattern, reflecting the increasing academic interest and relevance of ferroptosis research. This trend highlights the expanding impact of ferroptosis on understanding and addressing tumor resistance, establishing it as a rapidly advancing area of study within oncology. From 2014 to 2024, the number of publications in the field of ferroptosis and tumor resistance has shown an exponential growth trend. The calculated annual growth rate (AGR) is approximately 64.99%, indicating a rapid expansion of interest in this research domain. Moreover, linear regression analysis revealed a high coefficient of determination (R² = 0.893), confirming a strong linear upward trend in publication output over the past decade.

Furthermore, to assess the academic impact of different publication types, we stratified the 2,663 publications into research articles (n = 2,166) and review articles (n = 497). Citation analysis revealed that research articles received a total of 64,591 citations, with an average of 29.82 citations per article. In contrast, review articles accounted for 26,614 citations, averaging 53.56 citations per article. This finding indicates that although review articles constitute a smaller proportion of the literature, they tend to have a higher citation impact, highlighting their significance in shaping the academic discourse of ferroptosis and tumor resistance research. These results are illustrated in **Figure 2B**.

### 3.2 Contributions of countries and institutions

**Figure 3:**
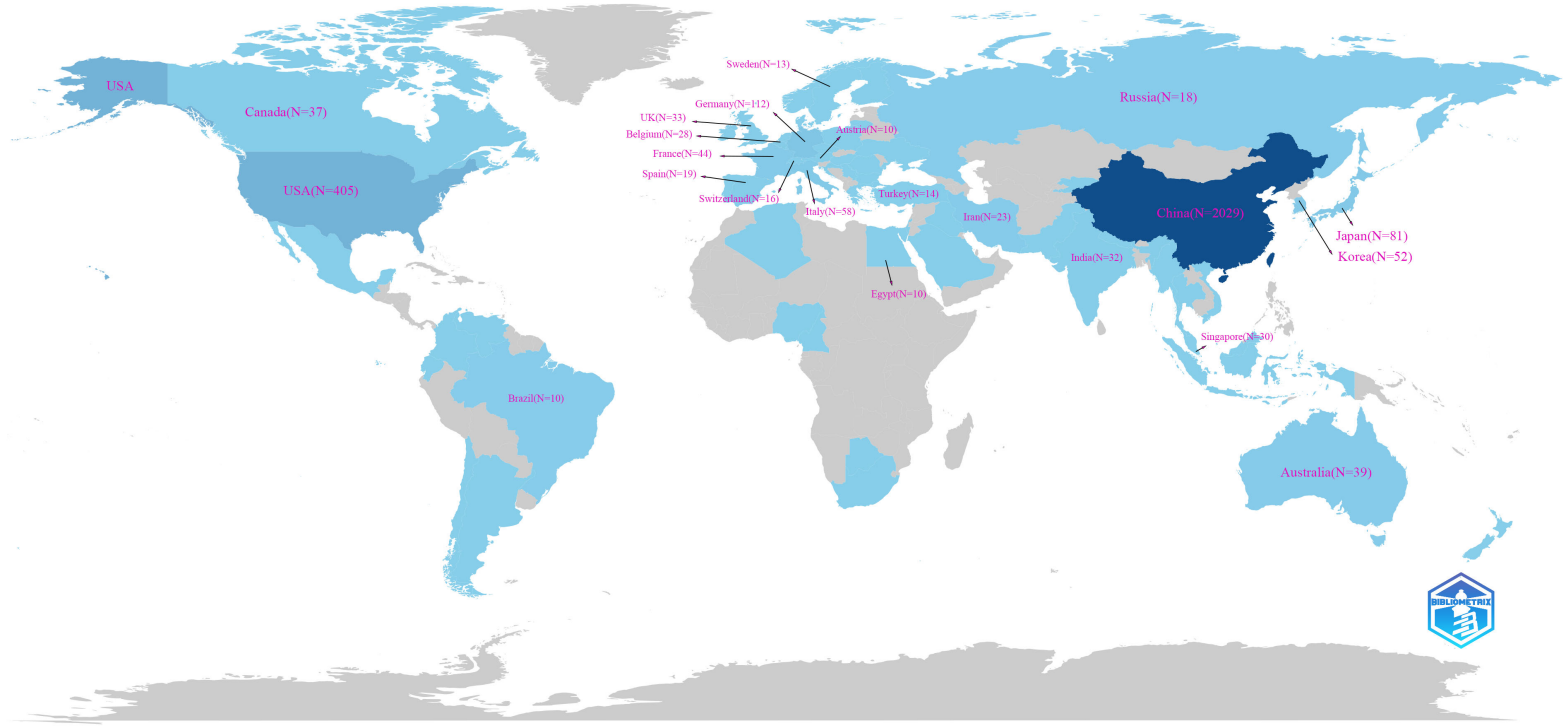
Global distribution of publications on ferroptosis and tumor resistance.

**Figure 4:**
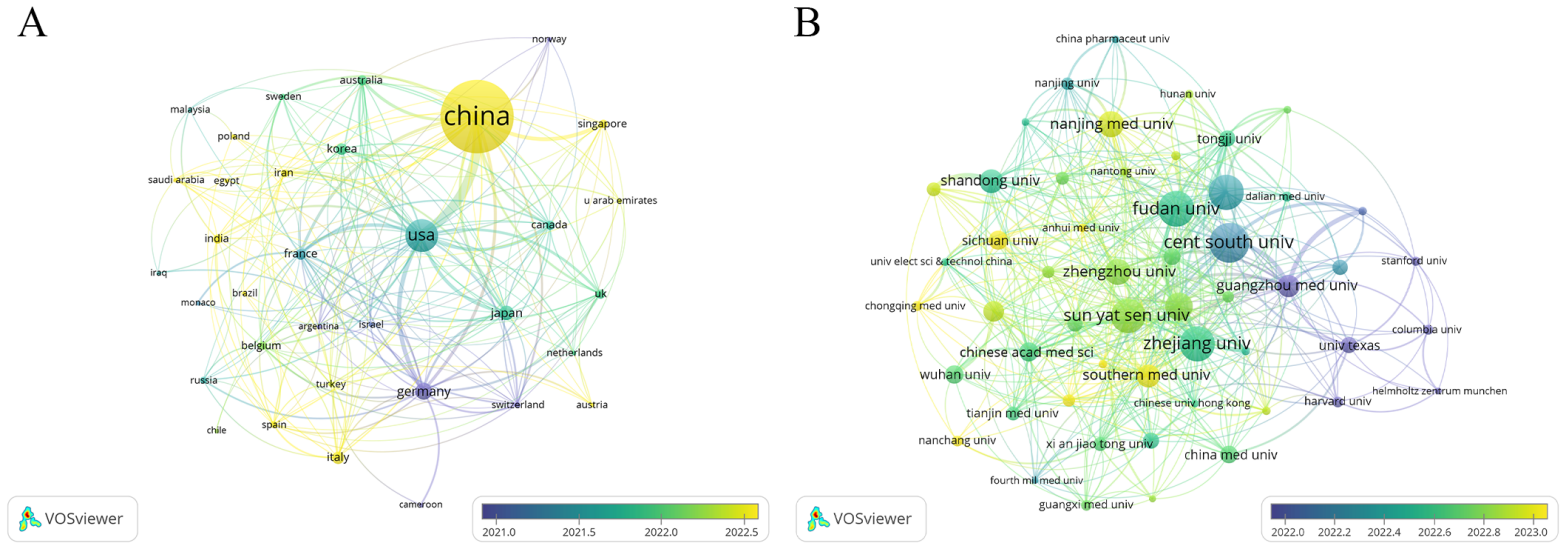
Collaboration networks in ferroptosis and tumor resistance research: **(A)** Country-level analysis and **(B)** Institution-level analysis.

A total of 65 countries and regions have contributed to the research on ferroptosis in tumor resistance. The global distribution of publications is presented in **Table 1** and **Figure 3**. China leads the field with 2,029 publications, accounting for 76.2% of the total output, followed by the United States (405 publications, 15.2%) and Germany (112 publications, 4.2%). In terms of citation impact, the United States and China rank first and second with 55,929 and 57,511 citations, respectively, while Germany ranks third with 20,570 citations. A collaboration network of 34 countries/regions with five or more publications was constructed (**Figure 4A**). In this network, the size of each node represents the publication volume, and the thickness of the connecting lines indicates the intensity of collaboration. The United States occupies a central position in the network, demonstrating strong collaborative ties with China and Japan. At the institutional level, 2,168 institutions were involved in this field. **Table 2** lists the top 20 institutions by publication volume. Central South University leads with 114 publications, followed by Fudan University (103 publications) and Shanghai Jiao Tong University (102 publications). Guangzhou Medical University (TLS = 205) and Zhejiang University (TLS = 212) exhibit the highest Total Link Strength, highlighting their active collaboration networks. **Figure 4B** visualizes the institutional collaboration network, showcasing institutions with more than 20 publications. In this network, node size corresponds to publication volume, line thickness indicates collaboration strength and node color reflects the temporal trend of research activity (from purple to yellow, representing earlier to more recent activity). The figure provides an intuitive overview of the major institutional collaborations within the field.

**Table 1:** Top 15 productive countries in ferroptosis and tumor resistance research.

Rank	Country	Documents	Citations	Total link strength
1	China	2029	57511	320
2	USA	405	55929	344
3	Germany	112	20570	139
4	Japan	81	9954	74
5	Italy	58	1188	39
6	Korea	52	3522	26
7	France	44	2296	88
8	Australia	39	5760	49
9	Canada	37	5049	61
10	UK	33	3290	50
11	India	32	782	32
12	Singapore	30	1268	39
13	Belgium	28	1530	39
14	Iran	23	382	27
15	Spain	19	228	37

**Table 2:** Top 20 productive organizations in ferroptosis and tumor resistance research.

Rank	Country	Organization	Documents	Citations	Total link strength
1	China	Cent South Univ	114	4843	192
2	China	Fudan Univ	103	3583	180
3	China	Shanghai Jiao Tong Univ	102	3038	185
4	China	Zhejiang Univ	102	3269	212
5	China	Sun Yat Sen Univ	98	3173	156
6	China	Chinese Acad Sci	79	2556	203
7	China	Zhengzhou Univ	75	1794	150
8	China	Nanjing Med Univ	74	1514	120
9	China	Shandong Univ	68	1864	135
10	China	Southern Med Univ	67	1169	135
11	China	Guangzhou Med Univ	65	9195	205
12	China	Huazhong Univ Sci & Technol	59	1321	112
13	China	Chinese Acad Med Sci	56	1503	119
14	China	Sichuan Univ	55	640	95
15	China	Wuhan Univ	54	1275	74
16	China	China Med Univ	50	1499	73
17	China	Wenzhou Med Univ	49	1669	96
18	China	Harbin Med Univ	47	1341	79
19	China	Tongji Univ	47	1479	90
20	USA	Univ Texas	47	11796	162

### 3.3 Authors and co-cited authors

**Figure 5:**
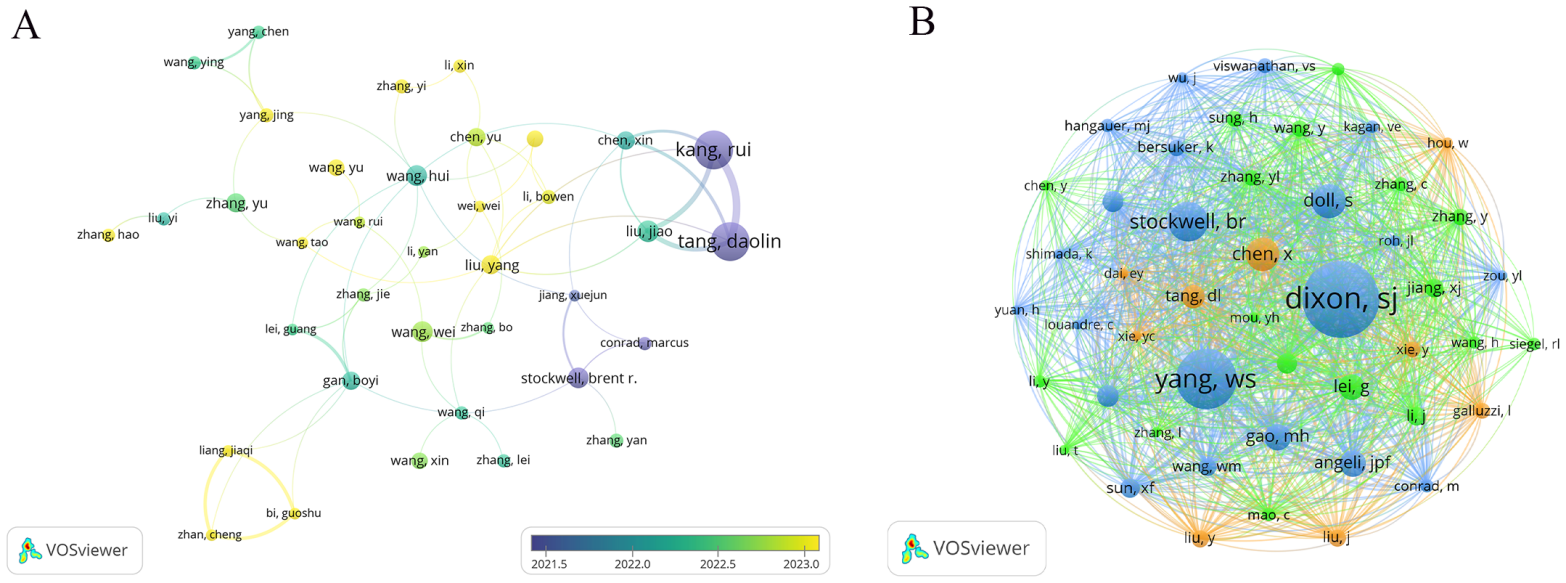
Author collaboration and co-citation networks in ferroptosis and tumor resistance research: **(A)** Author collaboration network and **(B)** Co-citation network.

This study identifies a total of 16,796 authors who have contributed to research on ferroptosis in tumor resistance. **Table 3** lists the top 15 prolific authors, with Kang Rui and Tang Daolin leading the field, each publishing 32 papers, accounting for 2.4% of the total publications. Stockwell Brent R. and Liu Jiao also stand out for their significant academic contributions, with 17 and 18 publications, respectively. Notably, Stockwell Brent R. ranks highest in citation impact, with his work cited 11,483 times, underscoring its pivotal role in advancing this field. **Figure 5A** visualizes the author collaboration network, encompassing 46 authors with at least 10 publications. The size of each node reflects the number of papers authored, while the connecting lines indicate the strength of collaborative relationships. The color of the nodes transitions from purple to yellow, representing the temporal evolution of research activity, with yellow nodes indicating authors who have been more active recently. The network reveals a core collaborative cluster formed by Kang Rui, Tang Daolin, Liu Jiao, Liu Yang, and Chen Xin, who have closely worked on the fundamental mechanisms of ferroptosis and its applications in tumor resistance. In addition, the co-citation analysis identifies 58,066 co-cited authors. **Table 3** highlights the 15 most frequently cited authors, with Dixon Scott J. emerging as the most frequently co-cited researcher in this domain. Dixon Scott J., Stockwell Brent R., Chen Xin, and Tang Daolin not only rank among the most co-cited authors but also are among the top contributors by publication count, further emphasizing their influence in the field. A co-citation network was constructed to explore the academic relationships among highly co-cited authors (**Figure 5B**). Among the co-cited authors, 48 have been co-cited more than 200 times. The node size in the network reflects the number of citations, while the connecting lines represent the frequency of two authors being co-cited, illustrating the intellectual connections within the field. Distinct clusters in the network represent major research directions and author groups. The blue cluster, centered on Dixon Scott J. and Yang Wen-Hsuan, focuses on fundamental theoretical mechanisms of ferroptosis, including metabolic regulation and cellular signaling pathways. The green cluster, led by Stockwell Brent R., emphasizes the application of ferroptosis in cancer therapy and its role in overcoming tumor resistance, acting as a bridge between basic research and clinical translation. The orange cluster, represented by Tang Daolin and Chen Xin, highlights research on ferroptosis-related biomarkers and the expansion of clinical applications.

**Table 3:** Top 15 authors and co-cited authors in ferroptosis and tumor resistance research.

Rank	Author	Documents	Citations	Co-cited author	Citations
1	Kang, Rui	32	3801	Dixon, Scott J.	2153
2	Tang, Daolin	32	3854	Yang, Wen-hsuan	1650
3	Liu, Jiao	18	819	Stockwell, Br	1030
4	Stockwell, Br	17	11483	Doll, Sebastian	864
5	Wang, Hui	17	1124	Chen, Xin	852
6	Wang, Wei	17	593	Gao, Minghui	664
7	Dixon, Scott J.	16	5214	Angeli, Jpf	648
8	Liu, Yang	16	181	Lei, Guang	633
9	Zhang, Yu	16	393	Tang, Daolin	577
10	Chen, Xin	15	1609	Hassannia, B	528
11	Chen, Yu	15	338	Jiang, Le	504
12	Gan, Boyi	15	2501	Koppula, Pranavi	481
13	Efferth, Thomas	14	952	Sun, Xiaofan	458
14	Roh, Jong-lyel	14	1565	Jiang, Xuejun	452
15	Wang, Xin	14	296	Li, Jie	444

### 3.4 Journal contribution and influence analysis

**Figure 6:**
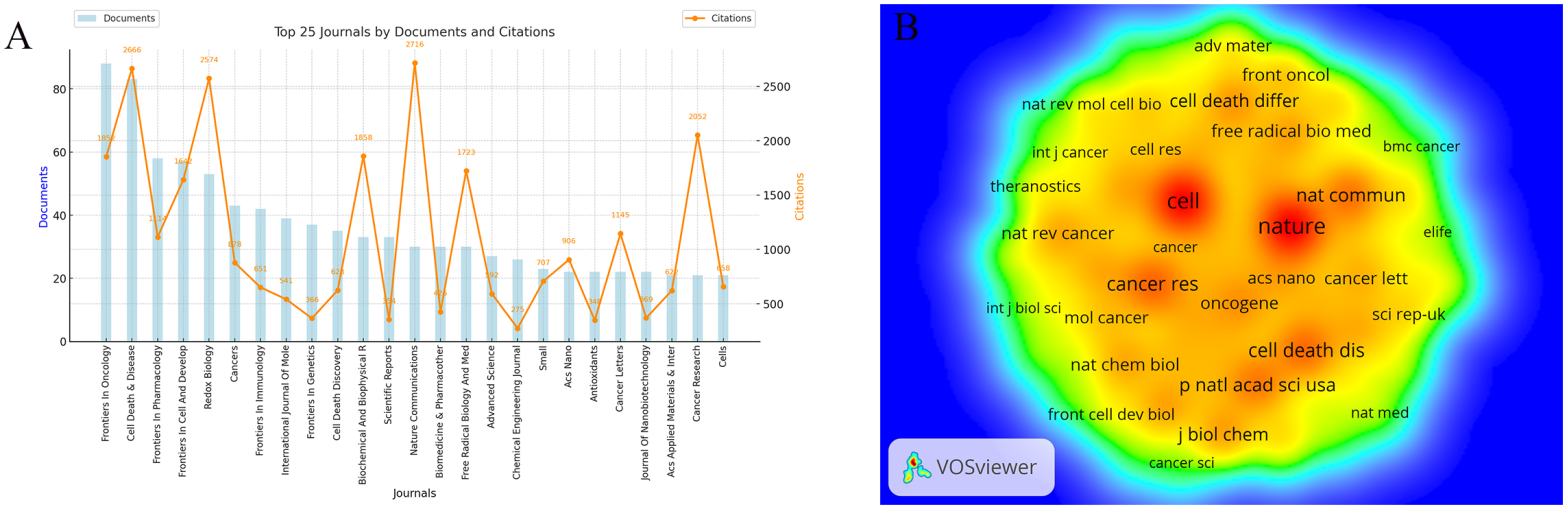
Analysis of Journals in Ferroptosis and Tumor Resistance Research: **(A)** Top 25 journals by documents and citations and **(B)** Co-citation network of core journals.

This study analyzed 2,663 publications on ferroptosis and tumor resistance, distributed across 560 academic journals. **Table 4** and **Figure 6A** present the top 25 journals ranked by publication volume and co-citation counts. In terms of publication volume, *Frontiers in Oncology* and *Cell Death & Disease* ranked highest, with 88 and 83 papers, respectively, followed by *Frontiers in Pharmacology* (58 papers), *Frontiers in Cell and Developmental Biology* (57 papers), and *Redox Biology* (53 papers). However, when considering citation impact, *Nature Communications* leads with 2,716 citations, followed by *Cell Death & Disease* (2,666 citations) and *Redox Biology* (2,574 citations), underscoring these journals’ significant academic influence. From the Journal Citation Reports (JCR) classification, 84% of the journals fall within the Q1 category, 12% in Q2, and 4% in Q3, indicating a predominance of high-impact journals in this research field. Among these, *ACS Nano* boasts the highest impact factor (15.8), followed by *Advanced Science* (14.3), *Chemical Engineering Journal* (13.3), and *Nature Communications* (14.7). The presence of such prestigious journals highlights the academic value and cutting-edge nature of research in this domain.

**Table 4:** Top 25 journals and co-cited journal in ferroptosis and tumor resistance research.

Journals	Documents	Citations	IF (2023)	JCR (2023)	Co-cited journals	Citations	IF (2023)	JCR (2023)
Frontiers In Oncology	88	1852	3.5	Q2	Nature	5881	50.5	Q1
Cell Death & Disease	83	2666	8.1	Q1	Cell	5713	45.6	Q1
Frontiers In Pharmacology	58	1114	4.4	Q1	Cancer Research	3234	12.5	Q1
Frontiers In Cell And Developmental Biology	57	1642	4.6	Q1	Cell Death & Disease	3170	8.1	Q1
Redox Biology	53	2574	10.7	Q1	Nature Communications	3019	14.7	Q1
Cancers	43	878	4.5	Q1	Proceedings Of The National Academy Of Sciences Of The United States Of America	2868	9.4	Q1
Frontiers In Immunology	42	651	5.7	Q1	Cell Death & Differentiation	2345	13.7	Q1
International Journal Of Molecular Sciences	39	541	4.9	Q1	Journal Of Biological Chemistry	2316	4	Q2
Frontiers In Genetics	37	366	2.8	Q2	Cancer Cell	2171	48.8	Q1
Cell Death Discovery	35	623	6.1	Q1	Free Radical Bio Med	2091	7.1	Q1
Biochemical And Biophysical Research Communications	33	1858	2.5	Q3	Oncogene	2040	6.9	Q1
Scientific Reports	33	354	3.8	Q1	Nature Reviews Cancer	2011	72.5	Q1
Biomedicine & Pharmacotherapy	30	426	6.9	Q1	Nature Chemical Biology	1968	12.9	Q1
Free Radical Biology And Medicine	30	1723	7.1	Q1	International Journal Of Molecular Sciences	1884	4.9	Q1
Nature Communications	30	2716	14.7	Q1	Redox Biology	1833	10.7	Q1
Advanced Science	27	592	14.3	Q1	Biochemical And Biophysical Research Communications	1831	2.5	Q3
Chemical Engineering Journal	26	275	13.3	Q1	Frontiers In Oncology	1790	3.5	Q2
Small	23	707	13	Q1	Cancer Letters	1700	9.1	Q1
Acs Nano	22	906	15.8	Q1	Acs Nano	1547	15.8	Q1
Antioxidants	22	348	6	Q1	Molecular Cell	1500	14.5	Q1
Cancer Letters	22	1145	9.1	Q1	Nature Cell Biology	1466	17.3	Q1
Journal Of Nanobiotechnology	22	369	10.6	Q1	Scientific Reports	1421	3.8	Q1
Acs Applied Materials & Interfaces	21	622	8.3	Q1	Cell Reports	1415	7.5	Q1
Cancer Research	21	2052	12.5	Q1	Molecular Cancer	1415	27.7	Q1
Cells	21	658	5.1	Q2	Science	1411	44.7	Q1

Co-citation analysis provides further insight into the academic standing of core journals in the field (**Figure 6B**, **Table 4**). *Nature* (5,881 co-citations) and *Cell* (5,713 co-citations) dominate in terms of co-citation counts, followed by *Cancer Research* (3,234), *Cell Death & Disease* (3,170), and *Nature Communications* (3,019). These high co-citation frequencies reflect their role as foundational resources for ferroptosis and tumor resistance research, widely recognized within the academic community. Notably, the high impact factors of *Nature* (50.5) and *Cell* (45.6) further emphasize their central roles as core references in this field.

**Figure 7:**
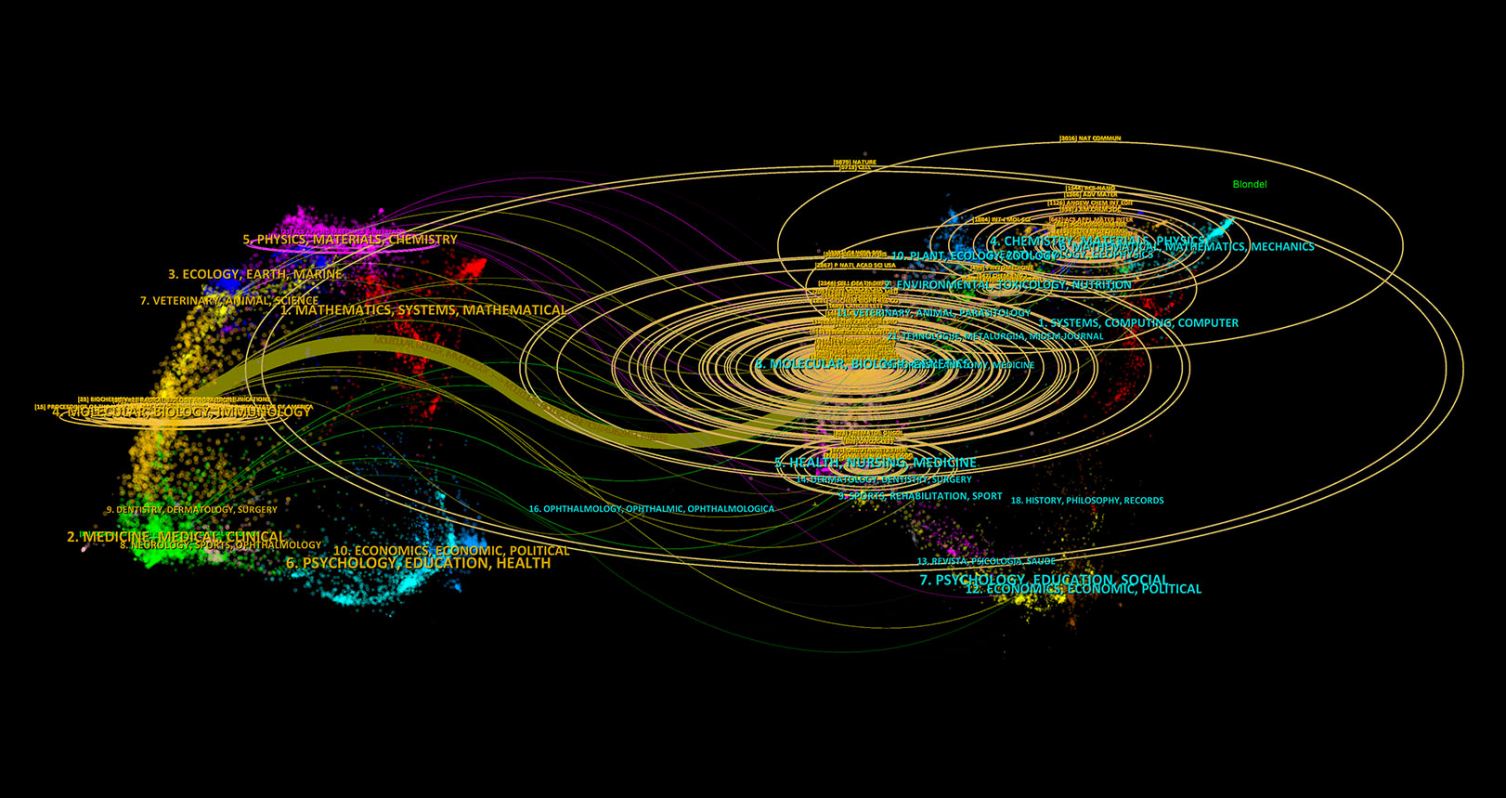
Dual-map overlay of citing and cited Journals in Ferroptosis and Tumor Resistance Research.

The dual-map overlay analysis (**Figure 7**) provides a comprehensive view of citation relationships across different thematic journals. The left side represents citing journals, which reflect the sources of current research, while the right side represents cited journals, illustrating the theoretical and data foundations of the field. The wave-like lines connecting the two sides indicate the interaction between research fronts and theoretical bases. The size of nodes represents the volume of publications in respective disciplines, and colors distinguish different academic fields. The analysis reveals that research on ferroptosis and tumor resistance predominantly spans molecular biology/genetics, medicine/clinical sciences, and ecology/biochemistry. The main citation pathways extend from molecular biology and medical journals to immunology and genetics journals, highlighting molecular biology and clinical medicine as the foundational pillars of this research domain, with immunology emerging as a critical area of recent development. The larger nodes of *Nature* and *Cell* on the right further underscore their significant academic influence and central role in the field. The clustering of cited journals on the right side of **Figure 7** demonstrates that the academic impact of research outputs is concentrated at the intersection of biological foundation studies and clinical medicine, reflecting the close integration of basic and applied research in ferroptosis and tumor resistance.

### 3.5 Analysis of references and co-cited references

Co-citation occurs when multiple articles are cited by the same paper, indicating a potential intellectual connection between the co-cited works. In this study, a total of 97,204 co-cited references were identified in the field of ferroptosis and tumor resistance. **Table 5** highlights the top 15 co-cited references. Among them, the most frequently co-cited article is *“Ferroptosis: an iron-dependent form of nonapoptotic cell death”* by Dixon SJ, published in *Cell* in 2012, with 1,379 co-citations and a total link strength (TLS) of 8,109. This seminal work laid the theoretical foundation for the ferroptosis research field. The second most co-cited reference is Dixon SJ’s 2014 article in *Cell*, *“Regulation of ferroptotic cancer cell death by GPX4”*, which has 886 co-citations and a TLS of 6,230, elucidating the critical role of *GPX4* in ferroptosis regulation. The temporal distribution of these highly co-cited references indicates that their publication dates predominantly range from 2012 to 2021, reflecting the rapid evolution of ferroptosis research over the past decade. Notably, these influential papers were published in top-tier journals such as *Nature Reviews Cancer*, *Nature*, and *Cancer Cell*, underscoring the high level of attention paid to ferroptosis within molecular biology, genetics, and oncology.

**Table 5:** Top 15 journals and co-cited journal in ferroptosis and tumor resistance research.

Rank	Reference	Journal	Published Year	Citations	Total link strength	Refs
1	Ferroptosis: an iron-dependent form of nonapoptotic cell death	Cell	2012	1379	8109	(1)
2	Regulation of ferroptotic cancer cell death by *GPX4*	Cell	2014	886	6230	(32)
3	Ferroptosis: A Regulated Cell Death Nexus Linking Metabolism, Redox Biology, and Disease	Cell	2017	638	4233	(73)
4	*ACSL4* dictates ferroptosis sensitivity by shaping cellular lipid composition	Nature Chemical Biology	2017	458	4055	(18)
5	Ferroptosis: mechanisms, biology and role in disease	Nature Reviews Molecular Cell Biology	2021	441	2720	(3)
6	Ferroptosis as a *p53*-mediated activity during tumour suppression	Nature	2015	422	3510	(34)
7	Targeting Ferroptosis to Iron Out Cancer	Cancer Cell	2019	408	3040	(74)
8	CD8+ T cells regulate tumour ferroptosis during cancer immunotherapy	Nature	2019	408	2837	(75)
9	Broadening horizons: the role of ferroptosis in cancer	Nature Reviews Clinical Oncology	2021	396	2663	(76)
10	The *CoQ* oxidoreductase *FSP1* acts parallel to *GPX4* to inhibit ferroptosis	Nature	2019	372	3654	(77)
11	*FSP1* is a glutathione-independent ferroptosis suppressor	Nature	2019	361	3648	(78)
12	Global Cancer Statistics 2020: GLOBOCAN Estimates of Incidence and Mortality Worldwide for 36 Cancers in 185 Countries	CA: A Cancer Journal for Clinicians	2021	356	1484	(79)
13	Dependency of a therapy-resistant state of cancer cells on a lipid peroxidase pathway	Nature	2017	351	2965	(80)
14	Ferroptosis: process and function	Cell Death and Differentiation	2016	318	2280	(81)
15	Pharmacological inhibition of cystine-glutamate exchange induces endoplasmic reticulum stress and ferroptosis	Elife	2014	315	2620	(82)

**Figure 8:**
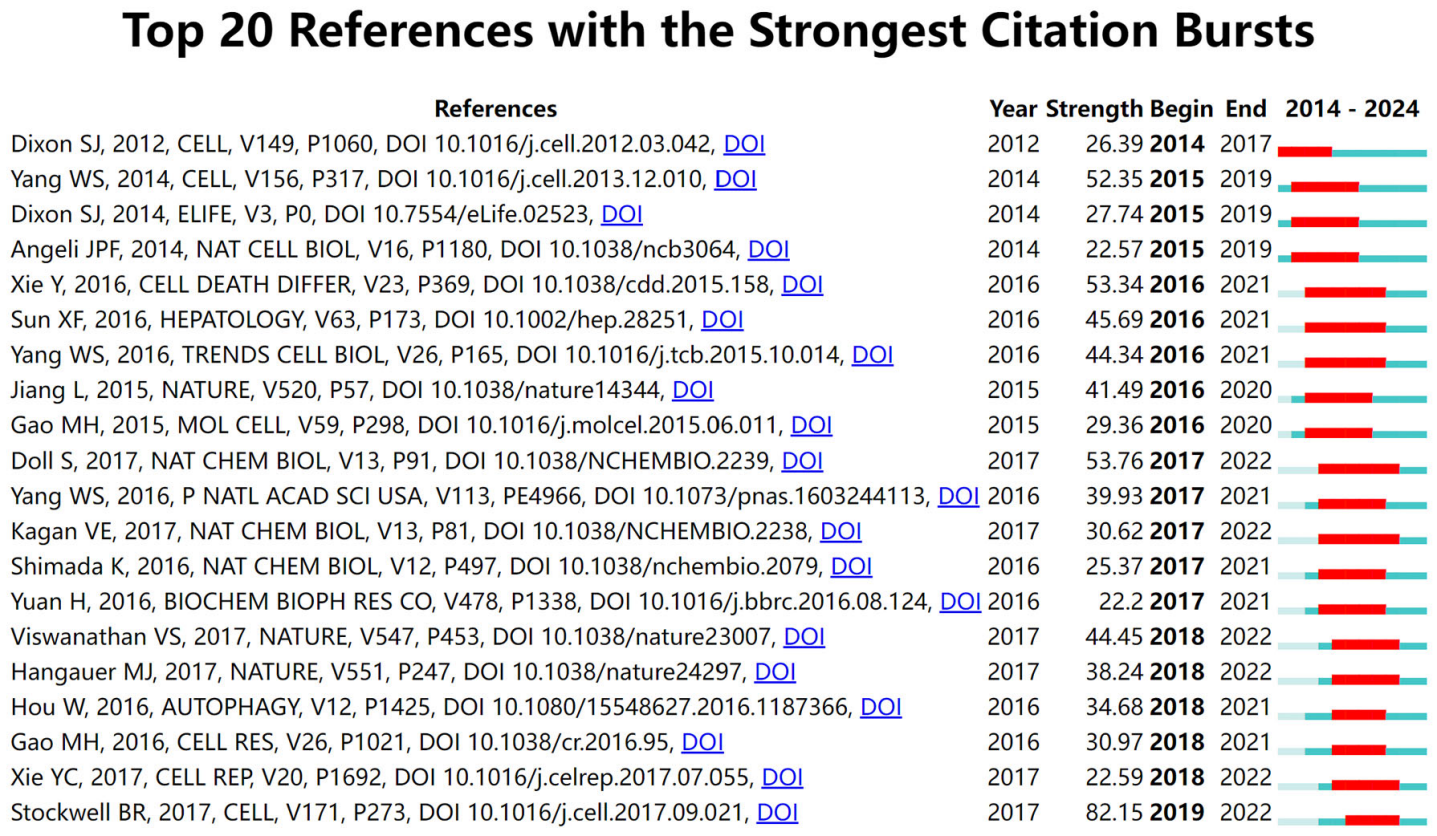
Top 20 references with the strongest citation bursts in ferroptosis and tumor resistance research.

Citation bursts, which denote a sudden surge in the frequency with which a reference is cited, highlight influential papers that have garnered significant academic attention within a short period. Using CiteSpace, the 20 references with the strongest citation bursts were identified (**Figure 8**). On the timeline, blue bars represent the publication period of a reference, while red bars indicate its burst period, reflecting when it experienced heightened citation activity. The reference with the highest burst strength (82.15) is Stockwell BR’s 2017 study published in *Cell*, which exhibited a citation burst from 2019 to 2022. This study provided pivotal insights into ferroptosis mechanisms and further solidified its role in cancer research.

### 3.6 Research hotspots revealed by keyword analysis

**Figure 9:**
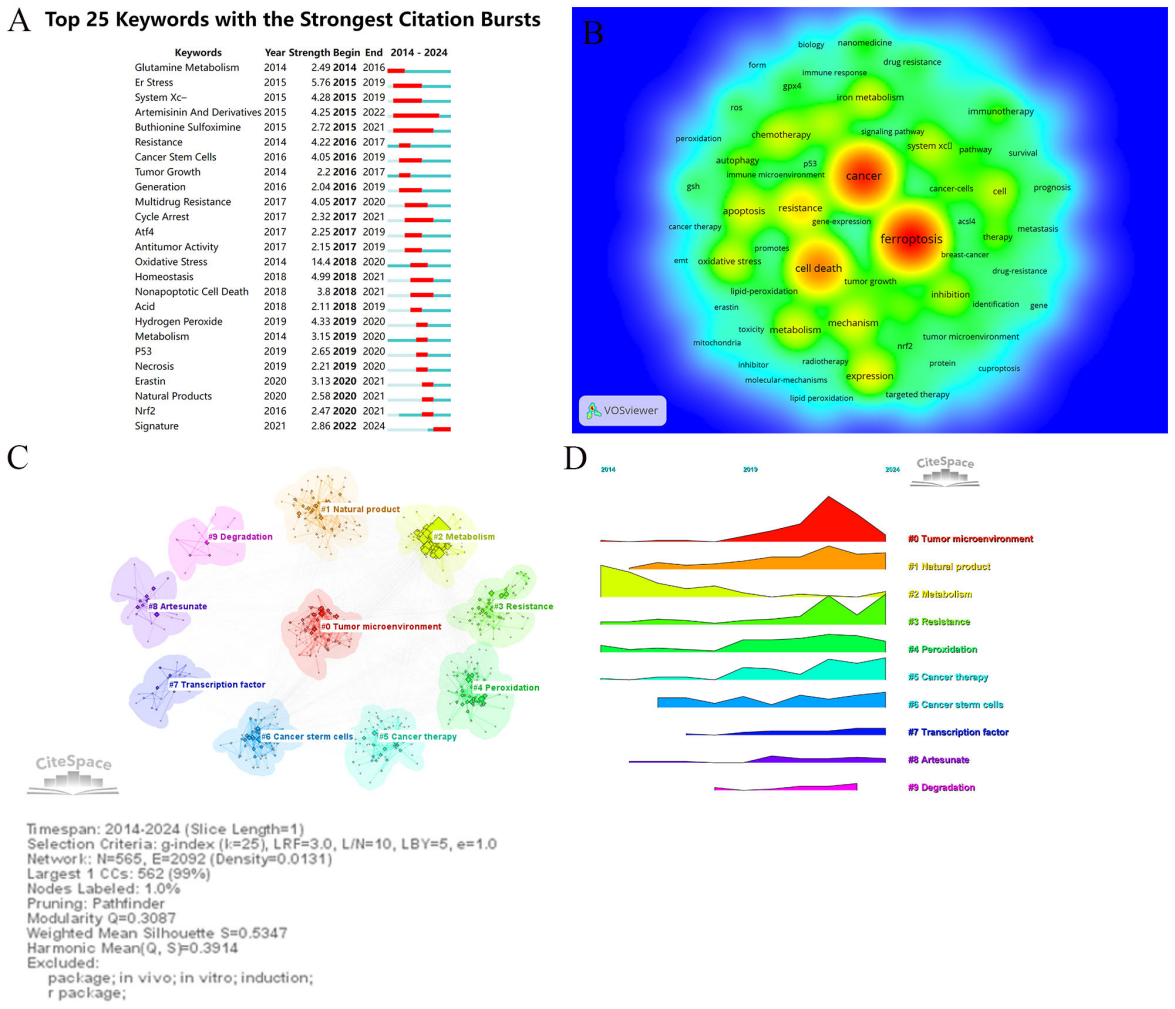
Keywords analysis in ferroptosis and tumor resistance research: **(A)** Top 25 keywords with the strongest citation bursts, **(B)** Keyword co-occurrence density map, **(C)** Keyword clustering analysis, and **(D)** Temporal trends of keyword clusters.

Keywords serve as vital indicators of research themes, helping to identify hotspots and developmental trends in a specific field. Using VOSviewer and CiteSpace, this study conducted an in-depth analysis of keywords in the ferroptosis and tumor resistance domain to uncover emerging trends and research trajectories. **Table 6** lists the top 25 high-frequency keywords in this field. The most frequently occurring keywords are “ferroptosis” (1,722 occurrences), “cancer” (1,483 occurrences), and “cell death” (516 occurrences), underscoring their central role in the research landscape. Other prominent keywords, such as “resistance” (474 occurrences), “oxidative stress” (313 occurrences), and “iron metabolism” (312 occurrences), highlight the critical importance of ferroptosis mechanisms in addressing tumor resistance. Keywords with significant citation bursts are summarized and visualized in **Table 6** and **Figure 9A**. Among these, “oxidative stress” exhibits the highest burst strength (14.4), followed by “ER stress” (5.76) and “system Xc⁻” (4.28). These findings indicate emerging research hotspots, particularly oxidative stress, whose citation burst extended to 2020, demonstrating its sustained relevance in ferroptosis research. **Figure 9B** presents a keyword co-occurrence density map, offering an intuitive visualization of interactions between keywords. Red areas represent concentrated research themes such as “ferroptosis,” “cancer,” and “resistance,” indicating their prominence in current research. Meanwhile, keywords like “immunotherapy,” “autophagy,” and “nanomedicine” appear in lower-density but growing regions, reflecting their expanding potential as emerging directions. Cluster analysis of keywords, conducted via CiteSpace, yielded 10 distinct clusters, each representing a unique research focus (**Figure 9C**). The most prominent cluster, “#0 Tumor microenvironment,” includes keywords such as “microenvironment,” “immune response,” and “inhibition,” highlighting the pivotal role of the TME in ferroptosis and resistance research. Cluster “#1 Natural product” points to the potential of plant-derived compounds and bioactive molecules in overcoming tumor resistance through ferroptosis modulation. Cluster “#2 Metabolism” emphasizes the role of iron, lipid, and amino acid metabolism in the regulation of ferroptosis. Cluster “#3 Resistance” covers multiple resistance types—including chemotherapy, radiotherapy, immunotherapy, and targeted therapy—focusing on key regulators like *GPX4* and *SLC7A11*. Cluster “#4 Peroxidation” emphasizes the importance of lipid peroxidation and oxidative damage in the initiation of ferroptosis and its role in sensitizing tumors to therapy. Cluster “#5 Cancer therapy” reflects growing interest in integrating ferroptosis into established oncologic treatments, such as chemotherapy and radiotherapy. Cluster “#6 Cancer stem cells” focuses on the vulnerability of tumor-initiating cells to ferroptosis, which may offer a new avenue to eliminate minimal residual disease and prevent relapse. Cluster “#7 Transcription factor” explores the regulation of ferroptosis-related genes via key transcriptional regulators, such as NRF2 and p53, highlighting molecular-level control mechanisms. Cluster “#8 Artesunate” indicates interest in specific ferroptosis-inducing agents—particularly artesunate, a derivative of artemisinin—with potential anticancer applications. Finally, Cluster “#9 Degradation” involves pathways and molecular processes related to protein or lipid degradation, suggesting novel links between ferroptosis and cellular homeostasis. Together, these clusters offer a comprehensive overview of the multifaceted landscape of ferroptosis research in tumor resistance, from mechanistic studies to translational and therapeutic applications.Temporal analysis of keyword clusters (**Figure 9D**) reveals dynamic changes in research focus. The modularity value (Q = 0.3087) indicates a meaningful clustering structure, and the silhouette score (S = 0.5347) reflects moderate clustering consistency, supporting the validity of the co-citation network analysis. TME-related studies have surged in prominence since 2019, reflecting its rapid emergence as a research hotspot. Conversely, clusters like “natural products” and “resistance” exhibit relatively steady research activity, signifying their foundational role in the field. Emerging clusters such as “peroxidation” and “cancer therapy” suggest potential future directions, demonstrating the expanding scope of ferroptosis research in addressing tumor resistance.

**Table 6:** Top keywords and citation bursts in ferroptosis and tumor resistance research.

Rank	Keyword	Occurrences	Total link strength	Keyword with the strongest citation burst	Burst
1	Ferroptosis	1722	8241	Oxidative Stress	14.3967
2	Cancer	1483	7009	Er Stress	5.7621
3	Cell Death	864	4718	Homeostasis	4.9868
4	Resistance	474	2692	Hydrogen Peroxide	4.3274
5	Expression	373	1973	System Xc–	4.2765
6	Mechanism	346	2006	Artemisinin And Derivatives	4.2519
7	Apoptosis	345	1878	Resistance	4.2215
8	Metabolism	332	1925	Multidrug Resistance	4.054
9	Oxidative Stress	313	1788	Cancer Stem Cells	4.0536
10	Iron Metabolism	312	1998	Nonapoptotic Cell Death	3.8005
11	Chemotherapy	305	1643	Metabolism	3.1526
12	System Xc–	282	1645	Erastin	3.132
13	Cell	275	1378	Signature	2.8647
14	Activation	268	1540	Buthionine Sulfoximine	2.7204
15	Inhibition	260	1469	P53	2.6506
16	Autophagy	213	1268	Natural Products	2.5813
17	Immunotherapy	203	1046	Glutamine Metabolism	2.4876
18	Nanomedicine	201	881	Nrf2	2.4654
19	Cancer-cells	188	1008	Cycle Arrest	2.319
20	Chemoresistance	186	1051	Atf4	2.2504
21	GPX4	176	983	Necrosis	2.2093
22	Therapy	175	937	Tumor Growth	2.2
23	Drug Resistance	171	989	Antitumor Activity	2.1458
24	Pathway	169	1016	Acid	2.1147
25	ROS	161	993	Generation	2.0399

## 4 Discussion

Cancer is a major global public health challenge, with its high incidence and mortality rates posing significant threats to human health and healthcare systems (27). Despite continuous advancements in conventional treatments such as chemotherapy and radiotherapy, as well as the rapid development of targeted therapies and immunotherapies that have significantly improved patient survival and quality of life, the long-term efficacy of cancer treatments remains severely hindered by drug resistance (28). Drug resistance, driven by the overexpression of MDR genes, metabolic adaptations to oxidative stress, and reprogramming of key signaling pathways, is a primary factor contributing to treatment failure. Overcoming drug resistance has become a critical challenge in the field of cancer therapy. In recent years, ferroptosis, a unique form of regulated cell death characterized by iron-dependent lipid peroxidation, has gained significant attention for its potential to overcome cancer drug resistance (16, 29). To comprehensively understand the current research landscape, hotspots, and future trends in ferroptosis within the context of cancer drug resistance, bibliometric analysis offers an effective approach. Bibliometric analysis uses quantitative methods to examine publication data, enabling the identification of research hotspots, development trends, and collaboration networks. This structured framework provides strategic insights for advancing scientific research. To systematically explore the role of ferroptosis in cancer drug resistance, this study analyzed publications indexed in the Web of Science Core Collection over the past decade. By leveraging bibliometric tools, this study represents the first comprehensive quantitative analysis of this topic. Key findings highlight the growth trends in publications, leading countries and institutions, core journals, influential authors, and the most-cited references in this domain.

### 4.1 Interpreting global research trends and institutional impact

Research on ferroptosis in the context of tumor resistance has undergone remarkable development over the past decade (**Figure 2**). From 2014 to 2018, this field was in its nascent stage, with annual publications increasing modestly from 6 to 29, and citations accumulating to 1,081. Although the research scale during this phase was limited, it laid a crucial foundation for the conceptualization and early exploration of ferroptosis. Between 2019 and 2021, the field experienced a rapid expansion, with annual publications surging to 282 and citations reaching 11,974. This period marked a significant broadening and deepening of research, as ferroptosis gained recognition as a promising therapeutic target for overcoming tumor resistance. Since 2022, the field has entered an accelerated phase of translational application, with annual publications climbing to 897 and citations surpassing 38,767. Research priorities have progressively shifted from basic mechanistic studies to clinical applications. The widespread adoption of high-throughput screening techniques and advancements in nanomedicine-based drug delivery systems have further catalyzed the integration of ferroptosis into tumor resistance research (30, 31). This trend reflects both academic enthusiasm and the growing clinical relevance of ferroptosis in overcoming tumor resistance. The expanding research scale provides a robust platform for basic studies, technological innovations, and translational efforts. This transition from foundational research to practical applications is poised to drive the development of novel therapeutic strategies for overcoming tumor resistance, paving the way for significant advancements in precision oncology.

This study reveals significant shifts in the global research landscape of ferroptosis in tumor resistance over recent years. China and the United States have emerged as central players in this field. With 2,029 publications, China leads in research productivity, accounting for 76.2% of total output, while the United States, despite contributing only 405 publications (15.2%), achieves 55,929 citations—nearly matching China’s 57,511 citations (**Figures 3**, **4A**). This indicates that U.S.-based research has higher academic influence, reflecting the quality and innovation of its studies. China has steadily ascended as a leader in ferroptosis research, making substantial progress in basic research and technological advancements. This growth is driven by robust government policies, significant funding allocations, and the rapid development of top-tier institutions such as Central South University, Fudan University, and Shanghai Jiao Tong University, which rank as the top three contributors (**Table 2**). The collaboration network (**Figure 4B**) further highlights the increasing activity of emerging institutions like Zhengzhou University and Huazhong University of Science and Technology, evidenced by their node colors transitioning from purple to yellow, signifying recent active contributions. Meanwhile, the United States maintains a pivotal position in international collaborations, underscoring its historical contributions to foundational mechanisms and methodological advancements in ferroptosis research. The University of Texas is the only non-Chinese institution in the top 20 by publication volume, with its citations significantly outpacing other institutions, underscoring its academic impact. European countries, including Germany, the United Kingdom, and France, demonstrate robust regional collaborations. This reflects Europe’s tradition of cross-institutional partnerships supported by comprehensive research policies. However, the declining size and brightness of nodes indicate a slight reduction in research momentum compared to earlier years. In contrast, the rapid growth of Chinese research suggests a potential shift in global research focus toward Asia. This transition could have profound implications for future research dynamics, as China’s expanding leadership combines high productivity with growing academic influence. The global research community can benefit from fostering deeper international collaborations to leverage these emerging trends and maintain the momentum in ferroptosis and tumor resistance research.

### 4.2 Mechanistic foundations and seminal contributions

We synthesized insights from highly influential and frequently cited publications to analyze their critical contributions to the advancement of ferroptosis research in the context of tumor drug resistance. High-citation studies are typically considered foundational and impactful within their respective fields. As illustrated in **Figure 8**, research on ferroptosis in tumor drug resistance has evolved through multiple phases, progressing from fundamental mechanistic studies to clinical translation. Each phase has been driven by key representative studies that shaped research trends and highlighted pivotal areas of focus. Between 2012 and 2016, foundational studies established the core mechanisms of ferroptosis. Dixon et al. (2012) first defined ferroptosis through experiments on RAS-mutant cancer cells, elucidating its iron-dependent lipid peroxidation mechanism, which laid the groundwork for subsequent studies (1). Yang et al. (2014) advanced the understanding of ferroptosis by identifying the critical regulatory role of *GPX4* in the ferroptotic process (32). Additionally, Angeli et al. (2014) demonstrated the regulatory role of ACSL4 in ferroptosis, revealing the crucial link between lipid metabolism and ferroptosis and establishing lipid metabolism as a central component of its molecular mechanism (33). From 2017 to 2019, research expanded to explore therapeutic applications of ferroptosis in cancer. Jiang et al. (2015) highlighted the potential of ferroptosis-inducing agents to overcome tumor drug resistance, providing direct evidence of its therapeutic promise (34). Gao et al. (2015) further supported this potential by demonstrating that combining ferroptosis inducers such as Erastin and RSL3 could enhance the efficacy of anti-tumor therapies (35). Similarly, Doll et al. (2017) investigated the molecular action of RSL3, showing that it induces ferroptosis through *GPX4* inhibition, demonstrating significant efficacy against drug-resistant tumors (18). These studies collectively propelled ferroptosis research from basic science to translational applications in oncology. Since 2020, ferroptosis research has increasingly expanded to integrate with immunotherapy and precision medicine. Lei et al. (2023) proposed that ferroptosis could synergize with immune checkpoint inhibitors, potentially improving the tumor immune microenvironment and providing a novel approach to overcoming immunotherapy resistance (36). Liu et al. (2023) demonstrated that nano-delivery systems significantly enhance the precision of ferroptosis-inducing agents while minimizing off-target toxicity (37). By incorporating multi-omics technologies, this research provides theoretical support and technical innovations for the precision treatment of drug-resistant tumors, establishing a robust foundation for the clinical translation of ferroptosis. Furthermore, Zhang et al. (2023) focused on oxidative stress regulation within the TME, demonstrating that targeting this microenvironment can enhance ferroptosis induction and further validate its central role in overcoming drug resistance (38).

### 4.3 Expanding horizons: from tumor microenvironment to nanomedicine

Keyword co-occurrence analysis provides valuable insights into the distribution and evolution of research hotspots within a specific field. In this study, core keywords identified include “Ferroptosis,” “Cancer,” “Resistance,” “Oxidative Stress,” “Metabolism,” and “System Xc⁻.” Emerging keywords such as “Tumor Microenvironment,” “Immunotherapy,” “Nanomedicine,” and “Autophagy” reflect the dynamic expansion of research frontiers in ferroptosis and tumor resistance. We utilized keyword co-occurrence, overlay visualization, temporal sequencing, and burst detection analyses to objectively identify research hotspots and emerging trends in this field. Using CiteSpace, these keywords were further subjected to clustering analysis, resulting in the identification of 10 distinct clusters. These clusters delineate the structural framework of ongoing research and highlight areas with significant potential for future exploration. The TME has emerged as one of the most prominent and highly active areas in keyword cluster analyses (#0 Tumor Microenvironment), underscoring its central role in ferroptosis research. As reflected in **Figure 9C**, the emergence of ‘tumor microenvironment’ as the largest keyword cluster further supports the observed research trend. In recent years, studies focusing on the TME have not only highlighted its pivotal role in tumor initiation, progression, and therapeutic resistance but also revealed its deep connection with ferroptosis regulation. From a temporal perspective, research on the TME has grown significantly since 2017, establishing it as a key research hotspot in recent years. The TME is a multifaceted ecosystem composed of tumor cells, immune cells, stromal cells, and other cell types, which coexist and engage in complex interactions that significantly influence tumor growth and progression (39). These interactions make the TME a critical platform for the regulation of ferroptosis, with its components and functions determining the initiation and effects of ferroptosis. Immunosuppressive cells in the TME, such as myeloid-derived suppressor cells (MDSCs) and M2 macrophages, play a dual role in regulating ferroptosis (15). On the one hand, these cells respond to lipid peroxidation byproducts induced by ferroptosis, such as damage-associated molecular patterns (DAMPs), further activating immunosuppressive signaling pathways and enhancing the immunosuppressive state within the TME. By secreting immunosuppressive factors, including IL-10 and TGF-β, they impair the function of effector immune cells, such as CD8+ T cells, significantly reducing the efficacy of immunotherapy (40, 41). Additionally, these cells upregulate antioxidant molecules such as PD-L1 and inhibit lipid peroxidation damage, thereby reducing tumor cell sensitivity to ferroptosis inducers such as Erastin and RSL3, further contributing to therapeutic resistance (42). On the other hand, these immunosuppressive cells secrete antioxidant factors, including glutathione and IL-10, directly modulating key molecular networks of ferroptosis (43). This mechanism involves the upregulation of antioxidant molecules such as *GPX4* and *SLC7A11*, which inhibit lipid peroxidation processes associated with ferroptosis and maintain cellular redox homeostasis. Together, these actions promote immune evasion and therapeutic resistance in tumors. Furthermore, studies have demonstrated that inflammatory signals induced by ferroptosis, such as the activation of the NF-κB pathway, can form a positive feedback loop, continuously recruiting more immunosuppressive cells into the TME, thereby reinforcing the immunosuppressive state. This feedback mechanism not only inhibits ferroptosis induction but also facilitates tumor progression, creating a vicious cycle of “immunosuppression-therapy resistance-ferroptosis inhibition” (44).

Stromal cells in the TME, particularly cancer-associated fibroblasts (CAFs), also play a critical role in ferroptosis regulation. CAFs enhance tumor cell survival by secreting pro-survival factors such as TGF-β. Furthermore, TGF-β1-activated CAFs promote tumor progression by modulating metabolic processes, such as autophagy, and specific molecules like FAP-α, potentially impacting ferroptosis pathways (45, 46). Notably, CAF-derived exosomes have been found to carry antioxidant molecules (e.g., miR-522) or regulatory signals that suppress the expression of lipid peroxidation-related genes (e.g., ALOX15), reducing the accumulation of lipid peroxidation products and thereby significantly mitigating ferroptosis effects and promoting chemoresistance in tumor cells (47). Metabolic reprogramming within the TME significantly enhances tumor cell resistance to ferroptosis. Through the upregulation of antioxidant metabolism pathways, such as *GPX4* and glutathione synthesis, and modulation of lipid metabolism, tumor cells adapt to environments with high iron levels and oxidative stress. For instance, the HIF-1α pathway activated in hypoxic environments not only promotes the expression of antioxidant genes but also enhances tumor cell survival by regulating iron distribution (48). These metabolic adaptations, combined with the heterogeneity of the TME, determine the dynamic characteristics of ferroptosis regulation. From a temporal perspective, the composition of the TME may undergo significant changes at different stages of ferroptosis induction. For example, effector immune cells exhibit higher activity in the early stages, whereas immunosuppressive cells may dominate in later stages (49). From a spatial perspective, hypoxic regions, characterized by specific iron metabolism features and the overexpression of antioxidant molecules, are often more resistant to ferroptosis, whereas oxidative stress regions are more susceptible (50, 51). This spatiotemporal dynamic suggests that distinct tumor regions may require differentiated therapeutic strategies. To enhance the antitumor efficacy of ferroptosis therapies, interventions targeting the TME have become a research focus. Reducing the number or function of immunosuppressive cells is a critical direction, such as employing CSF-1R inhibitors to decrease the abundance of M2 macrophages or CXCR2 antagonists to block MDSC migration. Additionally, disrupting the positive feedback loop of ferroptosis metabolic byproducts, such as lipid peroxidation products, can significantly weaken the immunosuppressive microenvironment. Strategies targeting stromal cells include using TGF-β signaling inhibitors to reduce CAF activity and disrupting tumor iron homeostasis by targeting iron metabolism-related molecules such as TfR1 and hepcidin. Moreover, normalizing vascular structures within the TME (e.g., VEGF inhibitors) or directly inhibiting HIF-1α activity can disrupt the hypoxic microenvironment and enhance ferroptosis induction efficiency. Future research should further explore the impact of TME dynamics on ferroptosis regulation and evaluate the clinical translational potential of combination therapeutic strategies across different tumor types.

According to keyword analysis, “oxidative stress” demonstrates the highest citation burst intensity (14.4) in ferroptosis research, with its research prominence steadily increasing until 2020 (**Figures 9A, B**). As a core driver of ferroptosis, oxidative stress is closely associated with high-frequency themes such as “ferroptosis,” “resistance,” and “cancer therapy.” Clustering analysis (**Figure 9C**) further highlights its strong connections with “lipid peroxidation” (Cluster #4) and “metabolic regulation” (Cluster #2). This underscores that oxidative stress is not only fundamental to the mechanisms of ferroptosis but also represents a critical node in the regulation of tumor resistance, showcasing its substantial research value and clinical translational potential. Ferroptosis is driven by oxidative stress-induced lipid peroxidation, where iron ions catalyze the generation of reactive oxygen species (ROS) via the Fenton reaction. ROS interact with lipid molecules, inducing peroxidation and leading to cell death. The antioxidant system plays a pivotal regulatory role in this process. Studies have shown that *GPX4* and *SLC7A11* are central factors in suppressing lipid peroxidation; their high expression significantly enhances the antioxidant capacity of tumor cells, enabling them to evade ferroptosis induced by immunotherapy, chemotherapy, or radiotherapy. Furthermore, tumor cells reprogram their metabolism to modulate oxidative stress balance and enhance resistance. For example, they upregulate glutathione (GSH) metabolism, activate *GPX4*, and regulate the Nrf2 signaling pathway to reduce ROS levels and mitigate lipid peroxidation accumulation. The keyword “System Xc−” also exhibits a high citation intensity (4.28), highlighting the importance of the glutamate-cysteine transport system in ferroptosis regulation. This system supplies cysteine to support GSH synthesis, effectively mitigating oxidative stress and protecting tumor cells from ferroptosis. Additionally, **Figure 9D** indicates that research on oxidative stress peaked after 2019, evolving from studies focused solely on molecular mechanisms to those exploring its interactions with the TME. Studies have demonstrated that targeting HIF-1α expression or alleviating hypoxia—such as through the use of capsaicin combined with ferroptosis inducers—can effectively suppress HIF-1α activity, mitigate hypoxic conditions, and enhance oxidative stress, thereby reversing tumor resistance and improving therapeutic outcomes (52). This dynamic feedback mechanism, termed “oxidative stress-antioxidant system-tumor resistance,” complicates the regulation of ferroptosis and poses significant challenges for clinical treatment. Future research should further investigate the spatiotemporal dynamics of oxidative stress, particularly its interactions with the TME. By dissecting the intricate mechanisms of hypoxia regulation, lipid metabolism, and antioxidant networks within the TME, and integrating personalized therapeutic strategies, ferroptosis research holds promise for developing more targeted solutions for treating therapy-resistant tumors.

Immunotherapy holds a significant position in the study of ferroptosis and tumor resistance. According to keyword analysis, “immunotherapy,” though ranked 18th in frequency, is among the top 25 keywords, indicating its importance in ferroptosis research and its emergence as a growing hotspot attracting increasing attention. Ferroptosis induces ICD, facilitating antigen release and immune-inflammatory responses, thereby providing a novel synergistic mechanism for immunotherapy. DAMPs released during ferroptosis, such as ATP, HMGB1, and calreticulin (CRT), are recognized by dendritic cells (DCs) and other antigen-presenting cells (APCs), which in turn activate CD8+ T cells and NK cells to mediate immune cytotoxic effects (53, 54). This mechanism is particularly critical for cold tumors, which lack immune-inflammatory cells. Ferroptosis, through the induction of antigen release and immune activation, can convert cold tumors into immunotherapy-responsive “hot tumors.” Studies have shown that a platinum(IV)–artesunate complex can enhance lipid peroxidation in the cytoplasm and mitochondria, triggering ferroptosis and releasing DAMPs associated with immune activation, thereby significantly enhancing immune responses and improving the treatment outcomes of cold tumors (55). The synergistic combination of immunotherapy and ferroptosis has demonstrated remarkable efficacy in overcoming tumor resistance. This strategy not only optimizes immune balance within the TME by reducing the activity of immunosuppressive cells but also significantly enhances the efficacy of immune checkpoint inhibitors (ICIs). This dual action endows immunotherapy with increased specificity and broad applicability under ferroptosis regulation, offering novel therapeutic approaches to address tumor resistance. Although research on immunotherapy in the context of ferroptosis and tumor resistance is still in its rapid development stage, it holds immense potential for future growth. Future studies should further investigate the synergistic mechanisms of immunotherapy and ferroptosis, particularly the optimal combinations of ferroptosis inducers and ICIs across various tumor types and therapeutic windows. Emphasis should also be placed on exploring cold tumors and highly resistant tumors, as well as unraveling the interactive dynamics between immunotherapy and TME. These efforts will provide a scientific foundation for personalized treatment strategies. By integrating the immunogenicity of ferroptosis with the specificity of immunotherapy, this field has the potential to deliver groundbreaking therapeutic strategies to overcome tumor resistance.

Nanomedicine has demonstrated unique advantages in the precise regulation of ferroptosis to overcome tumor resistance. As one of the top 25 keywords in the analysis (**Figures 9A, B**), nanomedicine is closely associated with ferroptosis, oxidative stress, and tumor resistance. In cluster analysis (Cluster #5), it is also highlighted as a research hotspot. The innovation of nanomedicine lies in its ability to significantly enhance the efficacy of ferroptosis inducers in the treatment of resistant tumors through targeted delivery and multimodal synergistic therapy. Targeted delivery is a major technical advantage of nanomedicine. Nanoparticles can be engineered with responsive materials, such as pH- or ROS-sensitive components, to ensure the precise delivery of ferroptosis inducers to tumor sites while minimizing nonspecific distribution in healthy tissues, thereby reducing systemic toxicity (56). For example, iron oxide nanoparticles leverage the high oxidative stress characteristics of the TME to release Fe³^+^, further amplifying oxidative stress, promoting lipid peroxidation, and inducing ferroptosis (57). This targeted delivery mechanism not only enhances the efficacy of ferroptosis but also significantly improves the safety of tumor treatments. Beyond single-drug delivery, nanoplatforms can serve as multifunctional carriers to support multimodal synergistic therapies. Nanomedicine exhibits remarkable technical advantages in the field of ferroptosis and tumor resistance by enabling the delivery of ferroptosis inducers and functioning as a versatile carrier for chemotherapeutic agents, targeted therapy drugs, and immunotherapeutics (58, 59). This multimodal approach precisely regulates ferroptosis pathways, effectively overcoming the limitations of conventional anticancer therapies in addressing tumor resistance. Tumor resistance is primarily driven by various mechanisms, including the dynamic balance of oxidative stress, metabolic reprogramming, drug efflux systems, and immunosuppressive states within the TME. Nanoparticles, with their efficient delivery and targeted release capabilities, can intervene in multiple resistance mechanisms simultaneously. For instance, ferroptosis inducers trigger lipid peroxidation and oxidative stress, directly disrupting tumor cell metabolic balance. Meanwhile, chemotherapeutic drugs (e.g., doxorubicin) exacerbate metabolic stress by impairing DNA repair mechanisms (60). Chemotherapeutics delivered via nanoparticles not only enhance ferroptosis induction but also reduce systemic toxicity by protecting normal cells through precise drug release. In targeted therapy, nanoparticles carrying tyrosine kinase inhibitors (TKIs, such as icotinib or erlotinib) can specifically inhibit signaling pathways driven by EGFR or BRAF mutations, while simultaneously sensitizing tumor cells to ferroptosis inducers (61, 62). Research has shown that tumor cells evade ferroptosis by upregulating antioxidant factors such as *GPX4*, *SLC7A11*, and *FSP1*. Nanoparticles loaded with gene-silencing materials or small-molecule inhibitors can directly target these antioxidant systems to reverse tumor resistance barriers (63, 64). This dual mechanism significantly enhances the therapeutic efficacy of ferroptosis inducers in multidrug-resistant tumors, such as lung cancer and melanoma. Additionally, the heterogeneity of the TME is a significant driver of tumor resistance. Hypoxic regions are often more resistant to chemotherapy and radiotherapy, and the dynamic changes in oxidative stress levels further affect ferroptosis induction efficiency. Nanoparticles with responsive materials (e.g., pH, ROS, or enzyme-responsive systems) can release drugs in a targeted manner and modulate redox balance within the TME, thereby overcoming the resistance of hypoxic regions to ferroptosis (65). Cluster and temporal trend analyses (**Figures 9C, D**) show that ferroptosis is closely linked with “oxidative stress,” “drug resistance,” and “microenvironment,” and nanomedicine emerges as a key approach to address these challenges. The unique advantage of nanomedicine lies in its ability to overcome the high toxicity and low targeting efficiency of traditional ferroptosis inducers while enhancing intervention in complex resistance mechanisms through combination therapy strategies. Temporal trends in keyword analysis (**Figure 9D**) indicate that nanomedicine has emerged as a growing hotspot in ferroptosis research, with increasing attention in recent years, underscoring its potential for clinical translation in precision medicine and overcoming tumor resistance. Future research should focus on optimizing the responsive design of nanomaterials and integrating multifunctional therapeutic modalities to achieve a deep synergy between ferroptosis and tumor resistance therapies, ultimately providing technical support for personalized treatment.

Recent studies have underscored the clinical potential of ferroptosis in overcoming tumor resistance and enhancing immunotherapy efficacy. For instance, Lin et al. (2025) demonstrated that targeting the circRNA cTRIP12 in pancreatic cancer enhances ferroptosis sensitivity and increases CD8^+^ T cell infiltration by regulating PD-L1 expression, offering a synergistic strategy with immune checkpoint inhibitors (66). In acute myeloid leukemia, Bruedigam et al. (2024) showed that the telomerase inhibitor imetelstat promotes ferroptosis via ACSL4- and FADS2-dependent lipid peroxidation, suggesting a potential for combination with oxidative stress-inducing therapies (67). These findings align with our bibliometric results highlighting “tumor microenvironment,” “immunotherapy,” and “resistance” as emerging hotspots, and emphasize the growing translational convergence of ferroptosis research and clinical oncology.

### 4.4 Limitations and future perspectives

Despite providing a comprehensive bibliometric analysis to uncover the current state and developmental trends in ferroptosis and tumor resistance research, this study has several limitations that should be acknowledged. First, the data for this study were exclusively sourced from the Web of Science Core Collection database, excluding other major academic databases such as PubMed, Scopus, and Embase. This limitation may result in the omission of certain high-quality studies and regional journals, potentially affecting the comprehensiveness and generalizability of the findings. Future research should consider integrating data from multiple databases to enhance the representativeness of the analysis. Second, this study primarily utilized bibliometric tools, such as VOSviewer and CiteSpace, which focus on quantitatively analyzing trends, research hotspots, and collaboration networks in academic publications. However, these tools lack the capacity for in-depth analysis of specific research content, such as experimental designs, methodological details, and outcomes. This may lead to the underestimation of the profound impact of certain high-quality studies. Additionally, the identification of research hotspots and trends heavily relies on publication and citation frequencies, which may limit the visibility of recently published high-quality articles with lower citation counts. In rapidly evolving fields like the intersection of ferroptosis and tumor resistance, cutting-edge topics may not yet be adequately reflected in keyword clustering and temporal trend analyses. This inherent time lag in bibliometric analysis is a notable limitation. Moreover, the dynamic nature of database updates may influence the results. Although the latest available dataset was used at the time of analysis, newly published articles on ferroptosis might not have been included due to the time gap between data collection and the completion of this study. Addressing this issue requires continuous updates to the research. Finally, there may be language or regional publication biases in certain research areas. For example, studies published in non-English languages or regional journals may not have been sufficiently covered, potentially limiting the global perspective of the analysis. Despite these limitations, the bibliometric trends identified in this study—such as the rising focus on the tumor microenvironment, nanomedicine, and immunotherapy—offer meaningful guidance for future research. By highlighting these evolving hotspots and their translational potential, the findings can help researchers prioritize directions with clinical relevance, foster interdisciplinary collaboration, and inform the development of novel therapeutic strategies targeting ferroptosis-related resistance mechanisms.

To further validate the reliability and clinical relevance of our bibliometric findings, we compared our results with recent review articles and meta-analyses. Zhang et al. (2024) highlighted the regulatory role of ferroptosis in EMT-driven tumor resistance and emphasized the importance of GPX4 and SLC7A11—mirroring our clustering results that identified “tumor microenvironment”, “resistance”, and these regulators as prominent themes (68). Nie et al. (2022) discussed how ferroptosis-related mechanisms, such as lipid peroxidation and amino acid metabolism, influence drug sensitivity and immunotherapy responses, reinforcing our findings related to “oxidative stress” and “chemoresistance” (69). Sun et al. (2023) revealed that androgen receptor variants in prostate cancer upregulate SLC7A11 and confer ferroptosis resistance, consistent with our identification of SLC7A11 as a central molecule in resistance regulation (70). Muluh et al. (2023) emphasized the emerging clinical value of ferroptosis in immunotherapy and multidrug resistance, consistent with our identification of “immunotherapy” and “resistance” as growing hotspots (71). Finally, a meta-analysis by Li et al. (2024) confirmed that ferroptosis-related gene signatures predict immunotherapy outcomes and patient prognosis across multiple cancer types (72). Collectively, these findings strongly support the translational potential of ferroptosis and align with the key hotspots identified in our bibliometric analysis.

## 5 Conclusion

This study is a comprehensive bibliometric analysis of ferroptosis in tumor resistance from 2014 to 2024. By systematically analyzing publication trends, global contributions, and research hotspots, this study elucidates the rapid development of ferroptosis research and its applications in overcoming tumor resistance. China has emerged as the leader in publication volume, while the United States demonstrates high academic influence. Recent studies highlight oxidative stress, tumor microenvironment, immunotherapy, and nanomedicine as pivotal research focuses in this field. Particularly, the integration of ferroptosis with oxidative stress regulation and immunotherapy strategies has shown promise in enhancing therapeutic efficacy. This study provides valuable insights into the research progress of ferroptosis and highlights key directions for future exploration.

## Data Availability

The raw data supporting the conclusions of this article will be made available by the authors, without undue reservation.

## References

[1] Dixon SJ Lemberg KM Lamprecht MR Skouta R Zaitsev EM Gleason CE . Ferroptosis: an iron-dependent form of nonapoptotic cell death. Cell. (2012) 149:1060–72. doi: 10.1016/j.cell.2012.03.042 PMC3367386 22632970

[2] Yang WS Stockwell BR . Synthetic lethal screening identifies compounds activating iron-dependent, nonapoptotic cell death in oncogenic-RAS-harboring cancer cells. Chem Biol. (2008) 15:234–45. doi: 10.1016/j.chembiol.2008.02.010 PMC2683762 18355723

[3] Jiang X Stockwell BR Conrad M . Ferroptosis: mechanisms, biology and role in disease. Nat Rev Mol Cell Biol. (2021) 22:266–82. doi: 10.1038/s41580-020-00324-8 PMC8142022 33495651

[4] Megyesfalvi Z Gay CM Popper H Pirker R Ostoros G Heeke S . Clinical insights into small cell lung cancer: Tumor heterogeneity, diagnosis, therapy, and future directions. CA Cancer J Clin. (2023) 73:620–52. doi: 10.3322/caac.21785 37329269

[5] Shriwas O Mohapatra P Mohanty S Dash R . The impact of m6A RNA modification in therapy resistance of cancer: implication in chemotherapy, radiotherapy, and immunotherapy. Front Oncol. (2020) 10:612337. doi: 10.3389/fonc.2020.612337 33718113 PMC7947626

[6] Hays E Bonavida B . YY1 regulates cancer cell immune resistance by modulating PD-L1 expression. Drug resistance updates: Rev commentaries antimicrobial Anticancer chemotherapy. (2019) 43:10–28. doi: 10.1016/j.drup.2019.04.001 31005030

[7] Vasseur S Guillaumond F . Lipids in cancer: a global view of the contribution of lipid pathways to metastatic formation and treatment resistance. Oncogenesis. (2022) 11:46. doi: 10.1038/s41389-022-00420-8 35945203 PMC9363460

[8] Lin Z Li J Zhang J Feng W Lu J Ma X . Metabolic reprogramming driven by IGF2BP3 promotes acquired resistance to EGFR inhibitors in non-small cell lung cancer. Cancer Res. (2023) 83:2187–207. doi: 10.1158/0008-5472.CAN-22-3059 37061993

[9] Guo X Tu P Zhu L Cheng C Jiang W Du C . Nanoenabled tumor energy metabolism disorder via sonodynamic therapy for multidrug resistance reversal and metastasis inhibition. ACS Appl materials interfaces. (2023) 15:309–26. doi: 10.1021/acsami.2c16278 36576435

[10] Wang Z Wang Y Li Z Xue W Hu S Kong X . Lipid metabolism as a target for cancer drug resistance: progress and prospects. Front Pharmacol. (2023) 14:1274335. doi: 10.3389/fphar.2023.1274335 37841917 PMC10571713

[11] Fu S Li Z Xiao L Hu W Zhang L Xie B . Glutamine synthetase promotes radiation resistance via facilitating nucleotide metabolism and subsequent DNA damage repair. Cell Rep. (2019) 28:1136–1143.e1134. doi: 10.1016/j.celrep.2019.07.002 31365859

[12] Hlouschek J Ritter V Wirsdörfer F Klein D Jendrossek V Matschke J . Targeting SLC25A10 alleviates improved antioxidant capacity and associated radioresistance of cancer cells induced by chronic-cycling hypoxia. Cancer Lett. (2018) 439:24–38. doi: 10.1016/j.canlet.2018.09.002 30205167

[13] Hu J Li Y Li H Shi F Xie L Zhao L . Targeting Epstein-Barr virus oncoprotein LMP1-mediated high oxidative stress suppresses EBV lytic reactivation and sensitizes tumors to radiation therapy. Theranostics. (2020) 10:11921–37. doi: 10.7150/thno.46006 PMC7667690 33204320

[14] Pérez-Ruiz E Melero I Kopecka J Sarmento-Ribeiro AB García-Aranda M De Las Rivas J . Cancer immunotherapy resistance based on immune checkpoints inhibitors: Targets, biomarkers, and remedies. Drug resistance updates: Rev commentaries antimicrobial Anticancer chemotherapy. (2020) 53:100718. doi: 10.1016/j.drup.2020.100718 32736034

[15] Kim R Taylor D Vonderheide RH Gabrilovich DI . Ferroptosis of immune cells in the tumor microenvironment. Trends Pharmacol Sci. (2023) 44:542–52. doi: 10.1016/j.tips.2023.06.005 37380530

[16] Zhang C Liu X Jin S Chen Y Guo R . Ferroptosis in cancer therapy: a novel approach to reversing drug resistance. Mol Cancer. (2022) 21:47. doi: 10.1186/s12943-022-01530-y 35151318 PMC8840702

[17] Ye L Jin F Kumar SK Dai Y . The mechanisms and therapeutic targets of ferroptosis in cancer. Expert Opin Ther Targets. (2021) 25:965–86. doi: 10.1080/14728222.2021.2011206 34821176

[18] Doll S Proneth B Tyurina YY Panzilius E Kobayashi S Ingold I . ACSL4 dictates ferroptosis sensitivity by shaping cellular lipid composition. Nat Chem Biol. (2017) 13:91–8. doi: 10.1038/nchembio.2239 PMC5610546 27842070

[19] Su J Zhao Q Zheng Z Wang H Bian C Meng L . Prospective application of ferroptosis in hypoxic cells for tumor radiotherapy. Antioxidants (Basel Switzerland). (2022) 1111:921. doi: 10.3390/antiox11050921 35624785 PMC9137794

[20] Zhao L Zhou X Xie F Zhang L Yan H Huang J . Ferroptosis in cancer and cancer immunotherapy. Cancer Commun (London England). (2022) 42:88–116. doi: 10.1002/cac2.12250 PMC8822596 35133083

[21] Lei G Mao C Yan Y Zhuang L Gan B . Ferroptosis, radiotherapy, and combination therapeutic strategies. Protein Cell. (2021) 12:836–57. doi: 10.1007/s13238-021-00841-y PMC8563889 33891303

[22] Yan R Xie E Li Y Li J Zhang Y Chi X . The structure of erastin-bound xCT-4F2hc complex reveals molecular mechanisms underlying erastin-induced ferroptosis. Cell Res. (2022) 32:687–90. doi: 10.1038/s41422-022-00642-w PMC9253326 35352032

[23] Wang X Shi W Li M Xin Y Jiang X . RSL3 sensitizes glioma cells to ionizing radiation by suppressing TGM2-dependent DNA damage repair and epithelial-mesenchymal transition. Redox Biol. (2024) 78:103438. doi: 10.1016/j.redox.2024.103438 39580966 PMC11625373

[24] Donthu N Kumar S Mukherjee D Pandey N Lim WM . How to conduct a bibliometric analysis: An overview and guidelines. J Business Res. (2021) 133:285–96. doi: 10.1016/j.jbusres.2021.04.070

[25] van Eck NJ Waltman L . Software survey: VOSviewer, a computer program for bibliometric mapping. Scientometrics. (2010) 84:523–38. doi: 10.1007/s11192-009-0146-3 PMC2883932 20585380

[26] Chen C . CiteSpace II: Detecting and visualizing emerging trends and transient patterns in scientific literature. J Am Soc Inf Sci Technol. (2005) 57:359–77. doi: 10.1002/asi.20317

[27] Bray F Laversanne M Sung H Ferlay J Siegel RL Soerjomataram I . Global cancer statistics 2022: GLOBOCAN estimates of incidence and mortality worldwide for 36 cancers in 185 countries. CA Cancer J Clin. (2024) 74:229–63. doi: 10.3322/caac.21834 38572751

[28] Xiang Y Liu X Wang Y Zheng D Meng Q Jiang L . Mechanisms of resistance to targeted therapy and immunotherapy in non-small cell lung cancer: promising strategies to overcoming challenges. Front Immunol. (2024) 15:1366260. doi: 10.3389/fimmu.2024.1366260 38655260 PMC11035781

[29] Lei G Zhuang L Gan B . The roles of ferroptosis in cancer: Tumor suppression, tumor microenvironment, and therapeutic interventions. Cancer Cell. (2024) 42:513–34. doi: 10.1016/j.ccell.2024.03.011 38593779

[30] Su Y Zhang Z Lee LTO Peng L Lu L He X . Amphiphilic Dendrimer Doping Enhanced pH-Sensitivity of Liposomal Vesicle for Effective Co-delivery toward Synergistic Ferroptosis-Apoptosis Therapy of Hepatocellular Carcinoma. Advanced healthcare materials. (2023) 12:e2202663. doi: 10.1002/adhm.202202663 36653312

[31] Yue R Zhou M Li X Xu L Lu C Dong Z . GSH/APE1 cascade-activated nanoplatform for imaging therapy resistance dynamics and enzyme-mediated adaptive ferroptosis. ACS nano. (2023) 17:13792–810. doi: 10.1021/acsnano.3c03443 37458417

[32] Yang WS SriRamaratnam R Welsch ME Shimada K Skouta R Viswanathan VS . Regulation of ferroptotic cancer cell death by GPX4. Cell. (2014) 156:317–31. doi: 10.1016/j.cell.2013.12.010 PMC4076414 24439385

[33] Friedmann Angeli JP Schneider M Proneth B Tyurina YY Tyurin VA Hammond VJ . Inactivation of the ferroptosis regulator Gpx4 triggers acute renal failure in mice. Nat Cell Biol. (2014) 16:1180–91. doi: 10.1038/ncb3064 PMC4894846 25402683

[34] Jiang L Kon N Li T Wang SJ Su T Hibshoosh H . Ferroptosis as a p53-mediated activity during tumour suppression. Nature. (2015) 520:57–62. doi: 10.1038/nature14344 25799988 PMC4455927

[35] Gao M Monian P Quadri N Ramasamy R Jiang X . Glutaminolysis and transferrin regulate ferroptosis. Mol Cell. (2015) 59:298–308. doi: 10.1016/j.molcel.2015.06.011 26166707 PMC4506736

[36] Lei H Li Q Pei Z Liu L Yang N Cheng L . Nonferrous ferroptosis inducer manganese molybdate nanoparticles to enhance tumor immunotherapy. Small (Weinheim an der Bergstrasse Germany). (2023) 19:e2303438. doi: 10.1002/smll.202303438 37420331

[37] Liu J Li X Chen J Zhang X Guo J Gu J . Arsenic-loaded biomimetic iron oxide nanoparticles for enhanced ferroptosis-inducing therapy of hepatocellular carcinoma. ACS Appl materials interfaces. (2023) 15:6260–73. doi: 10.1021/acsami.2c14962 36695492

[38] Zhang Y Du X He Z Gao S Ye L Ji J . A vanadium-based nanoplatform synergizing ferroptotic-like therapy with glucose metabolism intervention for enhanced cancer cell death and antitumor immunity. ACS nano. (2023) 17:11537–56. doi: 10.1021/acsnano.3c01527 37272777

[39] Cui K Wang K Huang Z . Ferroptosis and the tumor microenvironment. J Exp Clin Cancer Res. (2024) 43:315. doi: 10.1186/s13046-024-03235-0 39614322 PMC11607824

[40] Mahanti K Saha J Sarkar D Pramanik A Roy Chattopadhyay N Bhattacharyya S . Alteration of functionality and differentiation directed by changing gene expression patterns in myeloid-derived suppressor cells (MDSCs) in tumor microenvironment and bone marrow through early to terminal phase of tumor progression. J Leukoc Biol. (2024) 115:958–84. doi: 10.1093/jleuko/qiae013 38236200

[41] Bhardwaj V Ansell SM . Modulation of T-cell function by myeloid-derived suppressor cells in hematological Malignancies. Front Cell Dev Biol. (2023) 11:1129343. doi: 10.3389/fcell.2023.1129343 37091970 PMC10113446

[42] Zhao Y Du J Shen X . Targeting myeloid-derived suppressor cells in tumor immunotherapy: Current, future and beyond. Front Immunol. (2023) 14:1157537. doi: 10.3389/fimmu.2023.1157537 37006306 PMC10063857

[43] Mo W Liu S Zhao X Wei F Li Y Sheng X . ROS scavenging nanozyme modulates immunosuppression for sensitized cancer immunotherapy. Advanced healthcare materials. (2023) 12:e2300191. doi: 10.1002/adhm.202300191 37031357

[44] Cornice J Verzella D Arboretto P Vecchiotti D Capece D Zazzeroni F . NF-κB: governing macrophages in cancer. Genes. (2024) 15:197. doi: 10.3390/genes15020197 38397187 PMC10888451

[45] Chandra Jena B Sarkar S Rout L Mandal M . The transformation of cancer-associated fibroblasts: Current perspectives on the role of TGF-β in CAF mediated tumor progression and therapeutic resistance. Cancer Lett. (2021) 520:222–32. doi: 10.1016/j.canlet.2021.08.002 34363903

[46] Huang M Fu M Wang J Xia C Zhang H Xiong Y . TGF-β1-activated cancer-associated fibroblasts promote breast cancer invasion, metastasis and epithelial-mesenchymal transition by autophagy or overexpression of FAP-α. Biochem Pharmacol. (2021) 188:114527. doi: 10.1016/j.bcp.2021.114527 33741330

[47] Zhang H Deng T Liu R Ning T Yang H Liu D . CAF secreted miR-522 suppresses ferroptosis and promotes acquired chemo-resistance in gastric cancer. Mol Cancer. (2020) 19:43. doi: 10.1186/s12943-020-01168-8 32106859 PMC7045485

[48] Ghosh R Samanta P Sarkar R Biswas S Saha P Hajra S . Targeting HIF-1α by natural and synthetic compounds: A promising approach for anti-cancer therapeutics development. Molecules. (2022) 27:5192. doi: 10.3390/molecules27165192 36014432 PMC9413992

[49] Li C Hua K . Dissecting the single-cell transcriptome network of immune environment underlying cervical premalignant lesion, cervical cancer and metastatic lymph nodes. Front Immunol. (2022) 13:897366. doi: 10.3389/fimmu.2022.897366 35812401 PMC9263187

[50] Yang C Zhong ZF Wang SP Vong CT Yu B Wang YT . HIF-1: structure, biology and natural modulators. Chin J Natural Medicines. (2021) 19:521–7. doi: 10.1016/S1875-5364(21)60051-1 34247775

[51] Bilotta MT Antignani A Fitzgerald DJ . Managing the TME to improve the efficacy of cancer therapy. Front Immunol. (2022) 13:954992. doi: 10.3389/fimmu.2022.954992 36341428 PMC9630343

[52] Wang Y Zhou X Yao L Hu Q Liu H Zhao G . Capsaicin enhanced the efficacy of photodynamic therapy against osteosarcoma via a pro-death strategy by inducing ferroptosis and alleviating hypoxia. Small (Weinheim an der Bergstrasse Germany). (2024) 20:e2306916. doi: 10.1002/smll.202306916 38221813

[53] Efimova I Catanzaro E van der Meeren L Turubanova VD Hammad H Mishchenko TA . Vaccination with early ferroptotic cancer cells induces efficient antitumor immunity. J immunotherapy Cancer. (2020) 8:e001969. doi: 10.1136/jitc-2020-001369 PMC7668384 33188036

[54] Ding D Jiang X . Advances in immunogenic cell death for cancer immunotherapy. Small Methods. (2023) 7:e2300354. doi: 10.1002/smtd.202300354 37191336

[55] Fan R Deng A Lin R Zhang S Cheng C Zhuang J . A platinum(IV)-artesunate complex triggers ferroptosis by boosting cytoplasmic and mitochondrial lipid peroxidation to enhance tumor immunotherapy. MedComm. (2024) 5:e570. doi: 10.1002/mco2.v5.6 38774917 PMC11106517

[56] Fan D Cao Y Cao M Wang Y Cao Y Gong T . Nanomedicine in cancer therapy. Signal transduction targeted Ther. (2023) 8:293. doi: 10.1038/s41392-023-01536-y PMC10404590 37544972

[57] Liu Y Quan X Li J Huo J Li X Zhao Z . Liposomes embedded with PEGylated iron oxide nanoparticles enable ferroptosis and combination therapy in cancer. Natl Sci Rev. (2023) 10:nwac167. doi: 10.1093/nsr/nwac167 36684514 PMC9843134

[58] Hu T Gong H Xu J Huang Y Wu F He Z . Nanomedicines for overcoming cancer drug resistance. Pharmaceutics. (2022) 14:1606. doi: 10.3390/pharmaceutics14081606 36015232 PMC9412887

[59] Yin W Chang J Sun J Zhang T Zhao Y Li Y . Nanomedicine-mediated ferroptosis targeting strategies for synergistic cancer therapy. J materials Chem B. (2023) 11:1171–90. doi: 10.1039/D2TB02161G 36650960

[60] Abe K Ikeda M Ide T Tadokoro T Miyamoto HD Furusawa S . Doxorubicin causes ferroptosis and cardiotoxicity by intercalating into mitochondrial DNA and disrupting Alas1-dependent heme synthesis. Sci Signaling. (2022) 15:eabn8017. doi: 10.1126/scisignal.abn8017 36318618

[61] Kole E Jadhav K Singh R Mandpe S Abhang A Verma RK . Recent developments in tyrosine kinase inhibitor-based nanotherapeutics for EGFR-resistant non-small cell lung cancer. Curr Drug delivery. (2024) 22:249–60. doi: 10.2174/0115672018278617231207051907 38275043

[62] Zhang T Sun B Zhong C Xu K Wang Z Hofman P . Targeting histone deacetylase enhances the therapeutic effect of Erastin-induced ferroptosis in EGFR-activating mutant lung adenocarcinoma. Trans Lung Cancer Res. (2021) 10:1857–72. doi: 10.21037/tlcr-21-303 PMC8107764 34012798

[63] Tian X Zhang Y Zhang M Liu G Hao Y Liu W . Nanoparticles-encapsulated doxorubicin alleviates drug resistance of osteosarcoma via inducing ferroptosis. Nanotoxicology. (2024) 18:401–9. doi: 10.1080/17435390.2024.2369602 38907601

[64] Li K Lin C Li M Xu K He Y Mao Y . Multienzyme-like reactivity cooperatively impairs glutathione peroxidase 4 and ferroptosis suppressor protein 1 pathways in triple-negative breast cancer for sensitized ferroptosis therapy. ACS nano. (2022) 16:2381–98. doi: 10.1021/acsnano.1c08664 35041395

[65] Zhou W Jia Y Liu Y Chen Y Zhao P . Tumor microenvironment-based stimuli-responsive nanoparticles for controlled release of drugs in cancer therapy. Pharmaceutics. (2022) 14:2346. doi: 10.3390/pharmaceutics14112346 36365164 PMC9694300

[66] Lin H Zhu S Chen Y Lu J Xie C Liao C . Targeting cTRIP12 counteracts ferroptosis resistance and augments sensitivity to immunotherapy in pancreatic cancer. Drug resistance updates: Rev commentaries antimicrobial Anticancer chemotherapy. (2025) 81:101240. doi: 10.1016/j.drup.2025.101240 40154160

[67] Bruedigam C Porter AH Song A Vroeg In de Wei G Stoll T Straube J . Imetelstat-mediated alterations in fatty acid metabolism to induce ferroptosis as a therapeutic strategy for acute myeloid leukemia. Nat Cancer. (2024) 5:47–65. doi: 10.1038/s43018-023-00653-5 37904045 PMC10824665

[68] Zhang H Chen N Ding C Zhang H Liu D Liu S . Ferroptosis and EMT resistance in cancer: a comprehensive review of the interplay. Front Oncol. (2024) 14:1344290. doi: 10.3389/fonc.2024.1344290 38469234 PMC10926930

[69] Nie Z Chen M Gao Y Huang D Cao H Peng Y . Ferroptosis and tumor drug resistance: current status and major challenges. Front Pharmacol. (2022) 13:879317. doi: 10.3389/fphar.2022.879317 35668934 PMC9163417

[70] Sun R Yan B Li H Ding D Wang L Pang J . Androgen receptor variants confer castration resistance in prostate cancer by counteracting antiandrogen-induced ferroptosis. Cancer Res. (2023) 83:3192–204. doi: 10.1158/0008-5472.CAN-23-0285 PMC10543964 37527336

[71] Muluh TA Fu Q Ai X Wang C Chen W Zheng X . Targeting ferroptosis as an advance strategy in cancer therapy. Antioxidants Redox Signaling. (2024) 41:616–36. doi: 10.1089/ars.2024.0608 38959114

[72] Li S Tao K Yun H Yang J Meng Y Zhang F . Ferroptosis is a protective factor for the prognosis of cancer patients: a systematic review and meta-analysis. BMC Cancer. (2024) 24:604. doi: 10.1186/s12885-024-12369-5 38760742 PMC11102205

[73] Stockwell BR Friedmann Angeli JP Bayir H Bush AI Conrad M Dixon SJ . Ferroptosis: A regulated cell death nexus linking metabolism, redox biology, and disease. Cell. (2017) 171:273–85. doi: 10.1016/j.cell.2017.09.021 PMC5685180 28985560

[74] Hassannia B Vandenabeele P Vanden Berghe T . Targeting ferroptosis to iron out cancer. Cancer Cell. (2019) 35:830–49. doi: 10.1016/j.ccell.2019.04.002 31105042

[75] Wang W Green M Choi JE Gijón M Kennedy PD Johnson JK . CD8(+) T cells regulate tumour ferroptosis during cancer immunotherapy. Nature. (2019) 569:270–4. doi: 10.1038/s41586-019-1170-y PMC6533917 31043744

[76] Chen X Kang R Kroemer G Tang D . Broadening horizons: the role of ferroptosis in cancer. Nat Rev Clin Oncol. (2021) 18:280–96. doi: 10.1038/s41571-020-00462-0 33514910

[77] Bersuker K Hendricks JM Li Z Magtanong L Ford B Tang PH . The CoQ oxidoreductase FSP1 acts parallel to GPX4 to inhibit ferroptosis. Nature. (2019) 575:688–92. doi: 10.1038/s41586-019-1705-2 PMC6883167 31634900

[78] Doll S Freitas FP Shah R Aldrovandi M da Silva MC Ingold I . FSP1 is a glutathione-independent ferroptosis suppressor. Nature. (2019) 575:693–8. doi: 10.1038/s41586-019-1707-0 31634899

[79] Sung H Ferlay J Siegel RL Laversanne M Soerjomataram I Jemal A . Global cancer statistics 2020: GLOBOCAN estimates of incidence and mortality worldwide for 36 cancers in 185 countries. CA Cancer J Clin. (2021) 71:209–49. doi: 10.3322/caac.21660 33538338

[80] Viswanathan VS Ryan MJ Dhruv HD Gill S Eichhoff OM Seashore-Ludlow B . Dependency of a therapy-resistant state of cancer cells on a lipid peroxidase pathway. Nature. (2017) 547:453–7. doi: 10.1038/nature230070 PMC5667900 28678785

[81] Xie Y Hou W Song X Yu Y Huang J Sun X . Ferroptosis: process and function. Cell Death Differ. (2016) 23:369–79. doi: 10.1038/cdd.2015.158 PMC5072448 26794443

[82] Dixon SJ Patel DN Welsch M Skouta R Lee ED Hayano M . Pharmacological inhibition of cystine-glutamate exchange induces endoplasmic reticulum stress and ferroptosis. eLife. (2014) 3:e02523. doi: 10.7554/eLife.02523 24844246 PMC4054777

